# The formation of physician patient sharing networks in medicare: Exploring the effect of hospital affiliation

**DOI:** 10.1002/hec.3936

**Published:** 2019-10-28

**Authors:** Sebastian Linde

**Affiliations:** ^1^ Department of Economics, Seidman College of Business Grand Valley State University Allendale Michigan

**Keywords:** endogenous network formation, homophily, insurance networks, Medicare, physician patient sharing, unobserved degree heterogeneity

## Abstract

This study explores the forces that drive the formation of physician patient sharing networks. In particular, I examine the degree to which hospital affiliation drives physicians' sharing of Medicare patients. Using a revealed preference framework where observed network links are taken to be pairwise stable, I estimate the physicians' pair‐specific values using a tetrad maximum score estimator that is robust to the presence of unobserved physician specific characteristics. I also control for a number of potentially confounding patient sharing channels, such as (a) common physician group or hospital system affiliation, (b) physician homophily, (c) knowledge complementarity, (d) patient side considerations related to both geographic proximity and insurance network participation, and (e) spillover from other collaborations. Focusing on the Chicago hospital referral region, I find that shared hospital affiliation accounts for 36.5% of the average pair‐specific utility from a link. Implications for reducing care fragmentation are discussed.

## 1 INTRODUCTION

Recent work has helped uncover important relationships between the underlying structure of physician patient sharing networks and overall health care outcomes (Barnett, Song, & Landon, 2012a; Landon et al., 2018). One aspect of network structure that is related to health care outcomes is care fragmentation. Fragmented service delivery occurs when a patient is seen by a large number of physicians, introducing risk for lapses in coordination of care, with growing evidence that network structures with large numbers of specialist providers (i.e., higher levels of fragmented care) are associated with higher health care utilization and higher costs (Agha, Marzilli Ericson, Geissler, & Rebitzer, 2018; Agha, Frandsen, & Rebitzer, 2019). However, despite the advances in connecting physician patient sharing networks with outcome measures, less is known about the factors that give rise to these physician networks in the first place. By combining knowledge of why physicians seek certain ties with what we know about the impact of these structures on outcomes, the hope is that stakeholders seeking to improve the current medical system will be able to develop strategies that provide appropriate physician incentives for achieving the desired patient sharing network structure.

One hurdle in accomplishing this aim has been that establishing a credible case for causality in the presence of unobservable physician specific characteristics that may play a role in physician patient sharing (e.g., unobserved reputation or quality measures) is particularly problematic in social network settings where the researcher commonly works with cross sectional data on a sparse network that exhibits considerable degree heterogeneity (see, e.g., Chandrasekhar, 2016; Graham, 2017). 1A *sparse network* refers to a network where only a subset of all possible network links are present, whereas *degree heterogeneity* refers to the observation that some people within the network have several links, while others only have a few links. *Degree heterogeneity* is a function of both *observable* and *unobservable heterogeneities* in attributes across physicians. Because the sparsity of the network makes standard fixed effects approaches problematic within these settings, alternative methodologies have been developed for dealing with the identification issue introduced by unobserved degree heterogeneity (see, e.g., Graham, 2017; Kim, 2018). 2Pakes, Porter, Ho, and Ishii (2015) and Pakes (2010) provide a general framework for dealing with these types of identification issues more broadly in the context of estimation with moment inequalities. In the present study, I utilize a revealed preference framework that assumes that the observed physician patient sharing network is the outcome of a strategic network formation game (a la Jackson & Wolinsky, 1996), where the resulting network ties are assumed to be pairwise stable in that physicians do not want to deviate to either remove any existing ties or add any nonexisting ties. The equilibrium notion of pairwise stability provides me with inequalities that are used to estimate the parameters of physicians' link specific utility (using a tetrad maximum score estimator), which allows me to account for physicians' degree heterogeneity that may stem from physician‐specific unobservables.

Specifically, this study explores the degree to which physician patient sharing is driven by having a common hospital affiliation. Understanding the effect that a shared hospital affiliation has on physicians patient sharing behavior is of particular policy relevance as delineating accountable care organizations (ACOs) along the boundaries of hospital affiliated physicians has previously been proposed within the literature as a strategy for combating care fragmentation, and in turn, high costs and poor quality outcomes (Fisher, Staiger, Bynum, & Gottlieb, 2007). In order to attempt to capture a causal effect, I include a number of potentially confounding link formation channels in addition to controlling for physician‐specific unobservables. The included control variables draw from trends noted by Landon et al. (2012) and Barnett, Landon, O'Malley, Keating, and Christakis (2011) related to why physicians form collaboration relationships and share patients, including (a) institutional affiliations beyond a shared hospital affiliation; (b) physician homophily due to shared characteristics (e.g., gender and/or years of experience); (c) knowledge complementarity in terms of physician specialties; (d) patient considerations related to the geographic proximity and economic accessibility (based on insurance network inclusion) of other physicians; and (e) collaboration spillover, where physicians share patient groups due to existing collaboration ties and/or familiarity. Naturally, other physician idiosyncratic factors such as a physician's reputation for quality and service, his/her patient panel characteristics, and even personality, may factor into the number of patient sharing ties that one physician has when compared with another. As such, accounting for potential unobserved physician link heterogeneity is critical to evaluating the overall importance and relative significance of hospital affiliation on physicians' patient sharing.

The present study builds on, and contributes to, a number of different but related literatures. First, it contributes to the recent literature on empirical network formation estimation, where previous contributions have looked at airline networks (McCalman & Rysman, 2019) and social lending networks in India (Kim, 2018). The second is the literature on physician referrals, which has primarily focused on surveying physicians regarding their referral behavior (Barnett et al., 2011; Forrest, Nutting, Von Schrader, Rohde, & Starfield, 2006; Gonzalez & Rizzo, 1991; Kinchen, Cooper, Levine, Wang, & Powe, 2004). Although these studies provide important insights for factors that may contribute towards physicians' patient sharing, the present study differs by considering physician patient sharing (rather than direct referrals). Additionally, preferences for whom to collaborate with are elicited directly from shared patient data via a revealed preference approach rather than using survey data. The third related line of work has focused on using physician patient sharing data to approximate physician social/collaboration ties. Here, Barnett et al. (2011) and Landon et al. (2012) provided the important validation for this methodology, whereas other studies have followed, establishing relationships between observed network structures and health care outcomes in terms of utilization, cost, and quality (see, e.g., An, O'Malley, Rockmore, & Stock, 2018; Barnett et al., 2012b; Pollack, Weissman, Bekelman, Liao, & Armstrong, 2012, 2014) and to describe important properties of the physician patient sharing networks (Landon et al., 2018). The present study builds on, and contributes towards, this strand of work by focusing in on the physician incentives that give rise to the observed network structures studied within this literature, while paying close attention to the empirical issue of unobserved physician degree heterogeneity.

The dataset consists of 1,306 physicians (MD or DO) who practiced within the Chicago hospital referral region in 2016 and who collectively had 12,091 patient sharing ties. The physician level data are unusually rich in that it spans information on physician: Medicare patient sharing, characteristics, affiliations (group, hospital and system), geographic practice location, and insurance plan participation across most individual (under 65), small‐, mid‐, and large‐group market plans sold both on and off the federal and state exchanges as well as Medicare Advantage plans. Using these data, I find that physician patient sharing appears strongly motivated by common hospital affiliation. Additionally, although control variables also appear to provide utility to physician patient sharing ties, the relative importance of each of these channels is found to vary considerably.

The rest of this paper is organized as follows: Section 2 outlines the basic model. Section 3 presents the empirical specification along with the tetrad maximum score estimator and the main variable definitions. Section 4 covers the data and descriptives. Section 5 reports the main results, Section 6 provides a discussion of the main findings, and Section 7 concludes.

## 2 BASIC MODEL

Let *N*={1,…,*n*} be the set of physicians within market *m*, and let *i* and *j* denote typical members of the *N* set. The collaboration (or mutual patient sharing status) of all the physicians in market *m* is given by the network (or adjacency matrix) *g*={*g*_*i*,*j*_|*i*,*j*∈*N*}, where elements are *g*_*i*,*j*_=1 if *i* and *j* are collaborators, and *g*_*i*,*j*_=0 if they are not. 3As is standard, *g*_*i*,*i*_=0, ∀ *i*∈*N*. Let *g*+*g*_*ij*_ denote network *g* with a link added between *i* and *j*, and *g*−*g*_*ij*_ denote network *g* with a link removed between *i* and *j*. Define *N*(*i*;*g*)={*k*∈*N*|*g*_*i*,*k*_=1} as the set of agents with whom *i* has a mutual (two‐way) collaboration (i.e., a link). The utility of a physician from a given network configuration *g* is written as the summed benefit from all of his/her collaboration ties. That is,

(1) $$U_{i}(g)=\overset{N}{\underset{k\in N(i;g)}{\sum }}u_{ik},$$

where *u*_*ij*_ is the benefit *i* receives from having *j* as a collaborator (i.e., this is a link specific payoff). The marginal benefit from *i* and *j* forming a link is given by 4Regarding the notation, if *g*_*ij*_=1, then *U*_*i*_(*g*+*g*_*ij*_)=*U*_*i*_(*g*), and if *g*_*ij*_=0, then *U*_*i*_(*g*−*g*_*ij*_)=*U*_*i*_(*g*). This captures the notion that a link can either exist or not. Also note that link specific payoffs are assumed to be symmetric in that *u*_*ij*_=*u*_*ji*_.

(2) $$mu_{ij}=\left(U_{i}(g+g_{ij})+U_{j}(g+g_{ij})\right)-\left(U_{i}(g-g_{ij})+U_{j}(g-g_{ij})\right)=\left(\overset{N}{\underset{k\in N(i;g+g_{ij})}{\sum }}u_{ik}+\overset{N}{\underset{k\in N(j;g+g_{ij})}{\sum }}u_{jk}\right)-\left(\overset{N}{\underset{k\in N(i;g-g_{ij})}{\sum }}u_{ik}+\overset{N}{\underset{k\in N(j;g-g_{ij})}{\sum }}u_{jk}\right)=\left(u_{ij}+u_{ji}\right)=2u_{ij},$$

where 2*u*_*ij*_ captures the direct symmetric benefits that accrue to *i* and *j* from forming a direct link.

The equilibrium notion used is that of pairwise stability (due to Jackson & Wolinsky, 1996). This states that the physician patient sharing network *g* is *pairwise stable* if

(i) for every *g*_*i*,*j*_=1, *U*_*i*_(*g*)+*U*_*j*_(*g*) ≥ *U*_*i*_(*g*−*g*_*i*,*j*_)+*U*_*j*_(*g*−*g*_*i*,*j*_)⇔*mu*_*ij*_ ≥ 0; and
(ii) for every *g*_*i*,*j*_=0, *U*_*i*_(*g*)+*U*_*j*_(*g*) ≥ *U*_*i*_(*g*+*g*_*i*,*j*_)+*U*_*j*_(*g*+*g*_*i*,*j*_)⇔*mu*_*ij*_ ≤ 0.

Condition (i) states that if we observe a link between *i* and *j* (*g*_*i*,*j*_=1) then it must be the case that the overall payoff from having the link exceeds the payoff should *i* and *j* remove the link. Similarly, condition (ii) holds that if no link is observed between *i* and *j* (*g*_*i*,*j*_=0), then it must be the case that the joint payoff from not having a link is greater than that which would be obtained from *i* and *j* adding a link.

Next, we consider the empirical specification of the marginal utility and specify the tetrad inequalities that follow from our definition of pairwise stability and that will be used for the purpose of our parameter estimation.

## 3 EMPIRICAL APPROACH

### 3.1 Pair specific payoffs and tetrad inequality

In order to bring our model to data, we need to specify the pair specific payoff term, *mu*_*ij*_, and give it a functional form. 5Following prior work in this area, I employ a parsimonious specification where the marginal utility has a additively separable functional form (see, e.g., McCalman & Rysman, 2019, in the case of a network formation application or, Fox & Bajari, 2013, in a matching setting). To this end, let this marginal utility be given by

(3) $$mu_{ij}=\gamma HospitalAffiliation_{ij}+X_{ij}^{\prime }\beta +A_{i}+A_{j}+\xi_{ij},$$

where *Hospital Affiliation*_*ij*_ captures *i* and *j* ^*′*^ *s* degree of shared hospital affiliation; *X*_*ij*_ denotes the other pair specific attributes that are observed in the data; *A*_*i*_ and *A*_*j*_ are the unobserved physician‐level attributes (accounting for physician level degree heterogeneity), and *ξ*_*ij*_ is the pair‐specific error term that is assumed to be independently and identically distributed across all physician pairs. Identification rests on the assumption that *E*[*ξ*_*ij*_|*HospitalAffiliation*_*ij*_,*X*_*ij*_,*A*_*i*_,*A*_*j*_]=0.

As has previously been noted (see, e.g., Graham, 2017; Kim, 2018), the fact that the econometrician does not observe *A*_*i*_ or *A*_*j*_ poses an important threat to identification in the context of our problem, as omitting these physician fixed effects can result in biased estimates of our parameters *θ*=(*γ*,***β***). 6The issue stems from the fact that a given physician may have a lot of links due to some important unobserved characteristic—for example, being a highly regarded physician due to having a reputation of providing high quality care. This latent quality factor is hence captured by the *A*_*i*_ term.
^,^ 7Potential threats to identification include unobserved pair‐specific factors that are not additively separable, and therethrough not fully captured by the individual fixed effects. Further, although the approach does account for physician unobservables (*A*_*i*_,*A*_*j*_), our specification assumes these enter linearly and that they are not interacted with any of the pair‐specific characteristics, *X*_*ij*_. Additional details on study limitations are provided in Section 6, and estimates from an alternative specification are included within the Supporting Information as a robustness check.Moreover, the sparsity of real‐world social networks makes it problematic to estimate the unobserved fixed effects directly because most agents within these networks have few links (low degree). Alternative approaches for dealing with this issue have been suggested for inequality estimators within the matching (see, e.g., Fox, 2010) and network (Kim, 2018) literatures. 8Graham (2017) uses a similar approach to deal with unobserved degree heterogeneity within his tetrad Logit model. This approach has us select a tetrad of individuals—that is, four distinct individuals *i*,*j*,*k* and *l*, where *g*_*ij*_=1, *g*_*kl*_=1, *g*_*il*_=0, *g*_*jk*_=0, *g*_*ik*_=0, *g*_*jl*_=0. Here, pairwise stability implies that for the linked pairs (*g*_*ij*_=1, *g*_*kl*_=1), we have

(4) $$mu_{ij}\geq 0\;\wedge \;mu_{kl}\geq 0.$$

Whereas for the non‐linked pairs, we have (for *g*_*il*_=0, *g*_*jk*_=0)

(5) $$mu_{il}\leq 0\;\wedge \;mu_{jk}\leq 0,$$

and (for *g*_*ik*_=0, *g*_*jl*_=0):

(6) $$mu_{ik}\leq 0\;\wedge \;mu_{jl}\leq 0.$$

Combining inequalities (4) and (5) yields the tetrad inequality:

(7) $$mu_{ij}+mu_{kl}\geq mu_{il}+mu_{jk},$$

and similarly, combining (4) and (6) yields

(8) $$mu_{ij}+mu_{kl}\geq mu_{ik}+mu_{jl}.$$

From inequalities (7) and (8), it is clear that the individual level heterogeneity terms (*A*_*i*_,*A*_*j*_,*A*_*k*_,*A*_*l*_) will cancel out because they appear on both sides of the inequalities. As such, the omission of unobserved physician‐level characteristics that may cause degree heterogeneity within the data will not bias our parameter estimates.

### 3.2 Variable definitions

The physician pair specific variables (*Hospital Affiliation*_*ij*_,*X*_*ij*_) used within the empirical model control for a number of physician, patient, and institutional characteristics that have been identified within the literature as potential drivers of physicians' patient sharing. Here, I outline the variable definition for hospital affiliation and the controls for each of the other link formation channels.

#### 3.2.1 Hospital affiliation

To account for patient sharing that is motivated by shared hospital affiliation, I control for whether physicians *i* and *j* are members of a number of common hospitals. This is defined as $cSameHospital_{ij}=\left|H_{i}\cap H{}_{j}\right|$, which returns a count for the number of overlapping hospitals between *i* and *j* (i.e., the cardinality of the intersection between the set of physician hospitals of *i* (*H*_*i*_) and that of *j (H_j_)*).

#### 3.2.2 Other common institutional affiliations

It may also be the case that physicians within a particular group or system tend to share patients with other physicians within the boundaries of their shared organization. The measure for a shared physician practice is given by $cSamePractice_{ij}=\left|PP_{i}\cap PP_{j}\right|$, where *PP*_*i*_ denotes the set of practices that *i* is affiliated with. Shared system affiliation is captured using: $dSameSystem=1\left(\left|S_{i}\cap S{}_{j}\right|>0\right)$, where *S*_*i*_ denotes the set of hospital systems that *i* is affiliated with, and *dSameSystem* takes a value of 1 if *i* and *j* have at least one shared system and 0 otherwise.

#### 3.2.3 Homophily

Patient sharing may occur along social or friendship ties that are the result of physician homophily—that is, where physicians form patient sharing ties on the basis of their shared characteristics. To this end, I control for physician's gender and experience. I control for shared gender using *dSameGender*_*ij*_ = 1(*G*_*i*_ = *G*_*j*_, where *G*_*i*_ denotes the gender of *i* and 1(.) denotes an indicator function that takes the value of 1 when *i* and *j* have the same gender. For experience, the squared difference of physician *i* *′* *s* and *j* *′* *s* years of experience captures potential assortative link formation on the basis of shared years of experience: $sExperience_{ij}={\left(E_{i}-E_{j}\right)}^{2}$, where *E*_*i*_ denotes the years of experience of physician *i*.

#### 3.2.4 Knowledge complementarity

Patient sharing ties may be established based on physicians' differences in their expertise, that is, there may be important knowledge complementarities of certain physicians that drive patient sharing. To capture physicians' overlap in terms of their knowledge, I consider each physicians knowledge vector *K*_*i*_={*s*_*i*1_,*s*_*i*2_,…,*s*_*in*_}, where *s*_*i*1_=1 if physician *i* lists specialty *s*_*i*1_ as one of their specialties (and *s*_*i*1_=0 otherwise), and define *i* and *j* ^*′*^ *s* knowledge overlap as the uncentered correlation of *K*_*i*_ and *K*_*j*_: $jSpecialty_{ij}=K_{i}K_{j}^{\prime }/(\sqrt{K_{i}K_{i}^{\prime }}+\sqrt{K_{j}K_{j}^{\prime }})$. This measure takes values between 0 and 1, with 1 indicating perfectly overlapping specialties.

#### 3.2.5 Patient considerations

Patient sharing may be based on physicians' patient considerations. For example, distance may impose possible access to care issues for patients that the physician may wish to avoid if possible. To capture such considerations, I control for the distance between physician *i* and *j* *′* *s* practices using their zip codes to compute the travel distance. That is, distance is defined as *Distance*_*ij*_ = *d*(*Zipcode*_*i*_,*Zipcode*_*j*_), where *d*(.) measures the distance between the center of *Zipcode*_*i*_ and *Zipcode*_*j*_. Other patient considerations may relate to the patient's insurance in the cases where the Medicare patients have Medicare Advantage coverage. The influence of shared insurance network affiliation is captured by: $cSameMA\text{\_}Network{}_{ij}=\left|N_{i}^{MA}\cap N_{j}^{MA}\right|$, where $N_{i}^{MA}=\{Plan_{i1},Plan_{i2},\text{…},Plan_{ip}\}$ is a set of all Medicare Advantage plans that physician *i* is part of.

#### 3.2.6 Spillovers

Last, other professional interaction may lead physicians to share medicare patients. For example, it may be the case that physicians' sharing of other types of patient groups leads to the establishing of patient sharing ties that spillover and cause the physicians to also share Medicare patients. I investigate this possibility by controlling for physicians' shared participation in insurance networks for non‐Medicare (i.e., “other”) types of patients. This overlap is measured using $cSameOther\text{\_}Network_{ij}=\left|N_{i}^{O}\cap N_{j}^{O}\right|$, where $N_{i}^{O}=\{Plan_{i1}^{O},Plan_{i2}^{O},\text{…},Plan_{ip}^{O}\}$ is a set of all non‐Medicare Advantage plans that physician *i* is part of.

### 3.3 Tetrad maximum score estimation

The estimator employed within this study is a tetrad‐level maximum score estimator (a la Kim, 2018). 9Early work on the maximum score estimator was done by Manski (1975); however, more recent work by Fox (2018, 2010) has applied the maximum score estimator toward the estimation of pairwise‐stable two‐sided matching problems. This estimator has important computational advantages that make it feasible to apply it to large scale networks like the physician patient sharing network within this study. Moreover, Kim (2018) shows that the utility parameters are point identified, and importantly that bias concerns related to individual‐level unobserved heterogeneity are ameliorated by the fact that these individual fixed effects are canceled within the tetrad‐level maximum score estimator. The estimator in the case of one large market is given by

(9) $$Q(\beta )={\left(2\left(\begin{array}{c} N\\ 4 \end{array}\right)\right)}^{-1}\underset{i,j,k,l\in T}{\sum }\frac{1}{2}[1\left(s_{ijkl}(\theta )\geq s_{ikjl}(\theta )\right)+1\left(s_{ijkl}(\theta )\geq s_{iljk}(\theta )\right)].$$

In Equation (9), we have $s_{ijkl}(\theta )=1\left(X_{ij}^{\prime }\theta +X_{kl}^{\prime }\theta \right)\left(\left(1-a_{ik})(1-a_{il})(1-a_{jk})(1-a_{jl}\right)\right)$where *a*_*ik*_,*a*_*il*_,*a*_*jk*_,*a*_*jl*_∈*A*(*g*)=*G*+*I*, with *A*(*g*) denoting the adjusted adjacency matrix obtained by taking the actual adjacency matrix for network *g* (given by *G*) and adding an identity matrix to it so that the diagonal elements of *A*(*g*) are equal to one rather than zero. The adjustment term $\left(\left(1-a_{ik})(1-a_{il})(1-a_{jk})(1-a_{jl}\right)\right)$in *s*_*ijkl*_(*θ*) ensures that the tetrads selected satisfy the requirements for pairwise stability and also help ensure the existence of a pairwise stable equilibrium as well as identification (see Watts & Jackson, 2001, 2002; Kim, 2018). In terms of identification, as is common for this type of estimators, we are able to identify relative magnitudes but not an absolute level. As such, I impose a normalization where one of the *θ* parameters is set to ±1 causing all the other parameters to be interpreted relative to this value (see, e.g., Fox, 2010).

Estimation of the parameter vector (*θ*) is implemented using a differential evolution search algorithm, and confidence regions are computed using random subsampling (see, e.g., Fox & Santiago, 2014).

## 4 DATA AND DESCRIPTIVES

Focusing on the Chicago hospital referral region, I consider all physicians (with a MD or DO credentialing) that see Medicare patients and who are therefore present in the Centers for Medicare and Medicaid Services (CMS) Physician Compare registry in 2016. The Physician Compare dataset contains detailed physician level information regarding physician characteristics such as gender, years of experience, schooling, primary and secondary specialties, along with institutional information regarding physician group and hospital affiliations. Group practice and hospital affiliations are determined using Medicare claims data. A physician is defined as affiliated with a given hospital if they provided services there to at least three different patients, on at least three different occasions, occurring within the last year. In line with previous work (see, e.g., Barnett et al., 2012b), I remove physicians to whom referrals are less likely (i.e., physicians whose primary specialty are anesthesia, pathology, and radiology). The remaining physicians consist of 1,306 primary care physicians, medical, and surgical specialists. 10For a complete list of physicians specialties, see the Supporting Information. Additional physician level data on physicians' insurance network affiliations for both Medicare Advantage and other plans were sourced from the company Vericred. The non‐Medicare Advantage plans span most individual (under 65), small‐, mid‐, and large‐group market plans sold both on and off the federal and state exchanges. 11The insurance network data are for the 2016 plan period, whereas the Medicare Advantage network data is for 2017. Although the use of 2017 Medicare Advantage Network data might introduce some noise into the data, I use plan CMS provided crosswalks in order to map the 2017 plans back to 2016 plans. In so doing, I see that majority of the plans did not change between 2016 and 2017, with only about 3% of the plans being discontinued. These were excluded from the analysis. Hospital system affiliation was linked from the American Hospital Association.

Last, the physician level data are linked with CMS's data on physicians' sharing of Medicare patients. The patient sharing network ties are constructed from Medicare Claims data and were provided by Carelink Labs. Here, the unipartite physician network is a simple projection of a bipartite physician‐to‐patient network (as described in, e.g., Barnett et al., 2012b; Landon et al., 2013). Previous work has found that one would want a minimum of nine shared patients for a network link to be considered a shared patient tie and to further reduce the risk of including spurious links that may be driven by patient choice (Barnett et al., 2011). This notion of a minimum criterion for a link to denote a patient sharing tie is preserved within the data as per CMS privacy policies provider pairs who shared less than 11 distinct patients together (within the given time period) are not included in the data.

Table 1 presents summary statistics at the physician‐level. Here, we see that physicians are on average affiliated with 1.9 hospitals, 1 practice, and 0.8 systems. Close to 30% of the physicians are female and the physicians have on average 27.9 years of experience and 1.5 specialties. Last, looking at the physicians insurance network memberships, we see that they are on average part of 5.2 Medicare Advantage networks and 20.4 exchange (non Medicare‐Advantage) networks.

**Table 1:** Summary statistics at physician‐level

Variable	Mean	*SD*	*N*
cHospital	1.855	1.264	1,306
cPractice	1.048	0.560	1,306
cSystem	0.834	0.594	1,306
dFemale	0.298	0.457	1,306
cExperience_yrs	27.93	10.046	1,306
cSpecialties	1.467	0.636	1,306
cMA_Networks	5.172	5.852	1,306
cOther_Networks	20.382	8.199	1,306

*Note*. Prefixes “d” and “c” denote a dummy variable and a count variable measure, respectively.

Next, Table 2 provides summary statistics for the pair‐specific variables for three different cases: (i) the realized patient sharing ties within the data; (ii) the expected pair specific value from purely random link formation between any two physicians *i* and *j* within the data; and (iii) the expected pair values within a network where ties are formed randomly conditional on preserving the degree distribution of the realized patient sharing network. 12The conditionally random network is constructed using the monte carlo sampling method put forth by Viger and Latapy (2005). Comparing the values across the realized values and those obtained across random link formation provides details on the extent to which observed links between physicians are assortative. A consistent trend is that the realized average link value is higher than either one of the values expected from random link formation. This observation lends descriptive support toward physician collaboration ties being non‐random and instead the outcome of physicians assortative (and disassortative) sorting into patient sharing relationships.

**Table 2:** Summary statistics for physician‐pair variables

	(i) Realized links			(ii) Random links			(iii) Random links | degree dist.		
Variable	Mean	*SD*	*N*	Mean	*SD*	*N*	Mean	*SD*	*N*
cSameHospital	0.896	0.604	12,091	0.108	0.335	853,471	0.148	0.4	12,091
cSamePractice	0.521	0.630	12,091	0.060	0.266	853,471	0.078	0.302	12,091
dSameSystem	0.595	0.491	12,091	0.092	0.289	853,471	0.123	0.328	12,091
dSameGender	0.654	0.476	12,091	0.582	0.493	853,471	0.63	0.483	12,091
sExperience	190.290	253.587	12,091	202.171	264.447	853,471	203.608	264.138	12,091
jSpecialty	0.262	0.355	12,091	0.145	0.294	853,471	0.211	0.317	12,091
Distance_KM	5.286	8.403	12,086	11.302	8.411	852,165	11.946	8.861	12,086
cSameMA_Networks	1.843	3.046	12,091	1.324	2.658	853,471	1.269	2.437	12,091
cSameOther_Networks	15.127	6.689	12,091	12.804	6.788	853,471	13.721	6.123	12,091

*Note*. Prefixes “d,” “c,” “j,” and “s” denote a dummy variable, a count variable, an uncentered correlation, and a squared difference measure, respectively. Part (i) provides results from the patient sharing data; Part (ii) provides the expected results in the case of random link formation; and Part (iii) lists the results one would expect if link formation was random conditional on preserving the degree distribution within the observed data.

**Figure 1:**
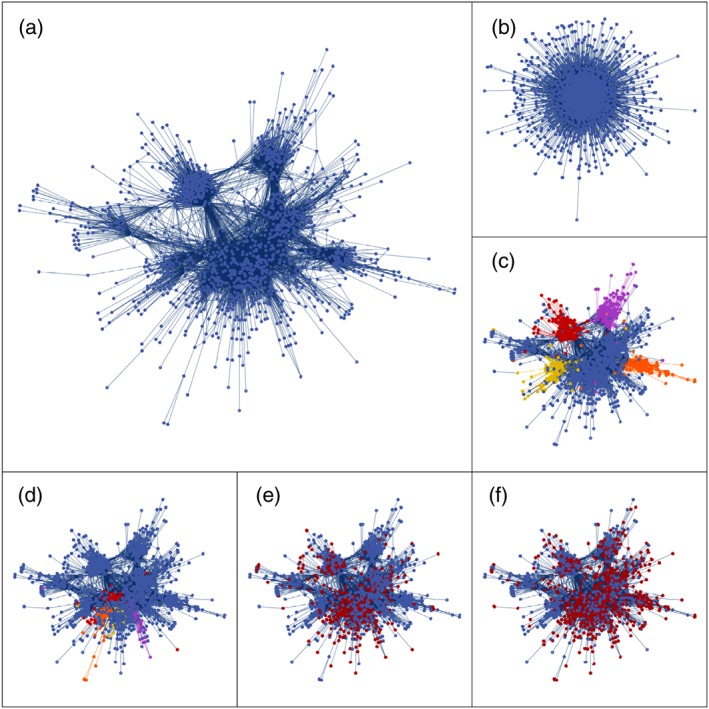
(a) Physician network (Chicago HRR); (b) random network with the same degree distribution as the physician network; (c) 4 hospitals highlighted; (d) hospital system highlighted; (e) Medicare Advantage insurance network highlighted; and (f) market‐based insurance plan highlighted [Colour figure can be viewed at http://wileyonlinelibrary.com]

The finding of assortative link formation can further be illustrated by mapping out the physician patient sharing network and visually examining it for evidence of non‐random network structures. This is done in Figure 1a where it showcases the physician network for the Chicago hospital referral region (HRR). In Figure 1a, we observe a number of clusters within the data, which again suggests non‐random link formations between physicians and considerable degree‐heterogeneity among physicians. Figure 1b illustrates the network structure one would expect if physicians were to form links at random conditional on the observed degree distribution within the data. Comparing Figure 1a,b, we are further able to appreciate the amount of structure that exists within the observed physician network. Next, Figure 1c shows the actual network (as in Figure 1a) but highlights physicians with hospital affiliations at Northwestern Memorial Hospital (red), University of Chicago Medical Center (purple), Rush (yellow) and University of Illinois hospital (orange). Figure 1d similarly highlights four hospitals that make up Presence hospital system in Chicago. What is clear in Figure 1c,d is that there appears to be clustering among physicians on the basis of hospital and hospital system affiliation. Figure 1e illustrates the set of physicians that are part of one of United Health's Medicare Advantage HMO plans within the data and highlight that this particular network has a significant existence within physicians affiliated with Presence hospital system and also among physicians associated with Northwestern Memorial but has hardly any physicians affiliated with the University of Chicago in its network. Last, for the sake of comparison, Figure 1f highlights physicians associated with a PPO silver plan offered by the Land of Lincoln Mutual Health Insurance Company on the exchange. This plan has a much wider coverage of physicians than the previously considered network.

The next section explores the relative effect of hospital affiliation on physicians' link specific utility by estimating the model put forth in Section 2.

## 5 RESULTS

Table 3 presents the tetrad maximum score point estimates along with their 95% confidence regions (column 1), a relative contribution of each average effect compared to the average effect of *dSameGender* (column 2), and the relative contribution of each effect when compared with the absolute link specific utility (column 3). 13The average absolute link utility is computed as: $\bar{u_{ij}}=\sum_{i}\left|\hat{\beta_{i}}\right|*\bar{x_{i}}$, where $\bar{x_{i}}$ denotes the average value of variable *x*_*i*_, $\left|.\right|$ is the absolute value, and the sum is taken across all variables. Using this as the reference utility ensures that the percentage contributions of all effects sum up to 100%. The estimates were obtained from a random subsample from the set of tetrads (consisting of 57,834 tetrads and 115,668 inequalities). The 95% confidence regions were constructed by subsampling using 200 draws, each with a 30% subsample of physicians (see, e.g., Fox & Santiago, 2014; Kim, 2018). Because the tetrad maximum score estimator is able to identify the relative, but not the absolute, magnitude of each marginal effect, I normalize the same gender dummy (*dSameGender*) to +1 and use it as the reference parameter. 14The parameter for *dSameGender* was set to +1 as this yielded a higher score (with more inequalities being satisfied) than a −1 value did.


**Table 3:** Maximum score estimates: A random sample 115,668 of all inequalities was used

			(2)	(3)
		(1)	Relative	% Absolute
Link channel	Variable	Estimate	Contribution	Contribution
*Hospital affiliations*	cSameHospital	34.063	46.7	36.5
		(11.612, 43.192)		
*Other affiliations*	cSamePractice	31.613	25.2	19.7
		(4.883 , 39.379)		
	dSameSystem	6.728	5.1	4.8
		(5.558, 8.7481)		
*Homophily*	dSameGender	1	1	0.8
		(−,−)		
	sExperience	−0.005	−1.5	1.1
		(−0.008 , 0.004)		
*Specialty*	jSpecialty	10.279	4.1	3.2
		(7.583, 13.751)		
*Patient considerations*	cSameMA_Networks	0.499	1.4	1.1
		(0.417, 0.694)		
	Distance_KM	−1.653	−13.4	10.5
		(−2.180, −1.466)		
*Spillovers*	cSameOther_Networks	1.231	28.5	22.3
		(1.058, 1.655)		
	Numb. Links	12,086	—	—
	Numb. Inequalities	115,668	—	—
	% Ineq. Satisfied	98.478	—	—

*Note*. A total of 95% confidence regions were constructed using 200 random draws of a 30% subsample of physicians. The same gender dummy (*dSameGender*) is normalized to +1 and it is used as the reference parameter estimate column. The average link utility effect relative to that of the average same gender effect is reported in the column (Relative Contribution). For example, the relative effect of *cSameHospital* is given by: $\frac{{\hat{\beta }}_{cSameHospital}*\bar{cSameHospital}}{{\hat{\beta }}_{dSameGender}*\bar{dSameGender}}=\frac{34.063*0.896}{1*0.654}=46.7$. The percentage contribution (% Absolute Contribution) of each effect is given by its relative absolute contribution towards the link specific utility obtained by multiplying the estimated parameter by the corresponding variable evaluated at its average population value, and dividing this by the overall link utility. For example, the percentage contribution of *cSameHospital* is given by: $\frac{\left|{\hat{\beta }}_{cSameHospital}\right|*\bar{cSameHospital}}{\sum_{i}\left|\hat{\beta_{i}}\right|*\bar{x_{i}}}=\frac{34.063*0.896}{83.6196}=0.365$, where $\bar{x_{i}}$ denotes the average value of variable *x*_*i*_.

The core hypothesis is that patient sharing will be driven by physicians having common hospital affiliations. I find support for this hypothesis as physicians belonging to one or more common hospitals (*cSameHospital*) positively contribute towards physicians' link specific utility. Looking at column 1 (Estimate) in Table 3, we see that the marginal effect of shared hospital affiliation (*cSameHospital*) is 34 times that of shared gender (*dSameGender*). 15This relative effect is computed by dividing the same hospital estimate by the same gender estimate, that is, $\frac{{\hat{\beta }}_{cSameHospital}}{{\hat{\beta }}_{dSameGender}}=\frac{34.063}{1}=34.063$. To gain further insight about the relative contribution of physicians' shared hospital affiliation toward their link specific utility, we can look at columns 2 and 3 in Table 3. Column 2 reports the average contribution of shared hospital affiliation relative to the average contribution of shared gender. In this case, the figure is 46.7 $\left(\frac{{\hat{\beta }}_{cSameHospital}*\bar{cSameHospital}}{{\hat{\beta }}_{dSameGender}*\bar{dSameGender}}=\frac{34.063*0.896}{1*0.654}=46.7\right)$ which tells us that for the average physician patient sharing link, shared hospital affiliation contributes 46.7 times more towards the link utility than does physicians' shared gender. Column 3 reports the percentage contribution of physicians' shared hospital affiliation relative to the absolute link specific utility. Here, we note that shared hospital affiliation on average contributes 36.5% $\left(\frac{\left|{\hat{\beta }}_{cSameHospital}\right|*\bar{cSameHospital}}{\sum_{i}\left|\hat{\beta_{i}}\right|*\bar{x_{i}}}=\frac{34.063*0.896}{83.6196}=0.365\right)$ to wards the absolute link specific utility.

Additional potential channels for patient sharing are further included as controls. Considerable variability in relative impact of these channels is noted. First, common physician practice groups (*cSamePractice*) also positively contribute to physician utility from link formation, as does belonging to a common hospital system (*dSameSystem*). Overall, motives due to these other shared institutional affiliations appear to contribute 24.5% of the absolute link specific utility.

Second, physician homophily is examined. Table 3 provides evidence of positive assortative link formation among physicians with a common gender (*dSameGender*) and years of experience (*sExperience*). Overall, homophily arguments are found to contribute a total of 1.9% of the absolute link specific utility.

Third, it was stated that physicians may form patient sharing ties on the basis of the complementarity of their knowledge. Table 3 shows that we in fact find the opposite result—physicians tend to form links with other physicians who share some degree of knowledge overlap with them (*jSpecialty*), and the benefit they derive from such a link appears to be increasing in the degree to which their skill sets overlap. In total, I find that link formation driven by knowledge overlap contributes 3.2% of the absolute link specific utility.

Fourth, physicians' patient considerations can influence who they share patients with. Specifically, the distance (*Distance_KM*) between two physicians offices negatively affects their link specific utility. An additional supporting finding is that physicians' link utility increases with the number of Medicare Advantage insurance plans (*cSameMA*_*Networks*) that they have in common. The complete contribution is here 11.6% of the absolute link specific utility.

Finally, sharing of a particular patient group was influenced by physicians' collaboration on other patient groups. This spillover effect was controlled for using the count for all other (non‐Medicare Advantage) insurance plans that the physicians had in common (*cSameOther*_*Networks*). The link specific utility is increasing in the number of insurance networks that the physicians share, and overall, I find that the contribution due to this spillover effect contributes 22.3% towards the absolute link specific utility.

## 6 DISCUSSION

In the present study, I apply a revealed preference framework to estimate physicians' link specific utility, using a rich dataset that allows me to focus on the effect of physicians' common hospital affiliation on patient sharing and to control for a number of other potentially confounding link formation channels, while also accounting for physician degree heterogeneity from latent physician characteristics. To summarize, I find considerable variation in the relative contribution of each channel towards the absolute link specific utility, where shared hospital affiliation contributes the most, followed by other institutional affiliations, collaboration spillovers, patient considerations, specialty/knowledge complementarity, and homophily. In what follows, I seek to discuss some of the implications of these results for administrators and policy makers and discuss limitations of the current study with potential for future work.

### 6.1 Institutional affiliation

The central finding of this paper is that shared hospital affiliation yields most of the link specific utility for physicians. This suggests that expanding physician and physician practice alignment with hospitals/hospital systems may be a potential strategy for reducing the fragmentation of patient care beyond current institutional boundaries (conditional on the number of links being the same). It is interesting to note that in the last couple of years, we have in fact observed such a trend with an increased number of physician practices aligning themselves with hospitals/hospital systems (see, e.g., Kane, 2018). From a patient perspective, it may suggest that selecting a physician within one affiliation network increases the likelihood of referral to a peer within the same practice, hospital, or health care system.

For the administrator, the finding that hospital affiliation plays a key role in physician patient sharing carries implications for minimizing patient “leakage,” which occurs when a patient leaves one institutional system to receive care from another. Managing and reducing patient leakage beyond the boundaries of physicians' core care team are of importance to both the reduction of patient care fragmentation but also to institutional bottom lines. The results found here suggest that physician group alignment with a hospital or institutional system may increase within system patient sharing, resulting in a move towards within system collaboration that occurs organically. These findings are consistent with those reported by Carlin, Feldman, and Dowd (2016) and Walden (2016), where the authors demonstrate that physicians under common ownership begin to refer to one another following a hospital merger.

From a policy point of view, the results reported here can be beneficial in the move towards defining Accountable Care Organizations. Prior work has suggested that using physician patient sharing data to select ACOs can possibly lead to better adoption by tapping into already existing network infrastructure among physicians (Landon et al., 2013). The finding that shared hospital affiliation contributes the most towards physician's link specific utility suggests that forming ACOs around the institutional boundaries of hospitals can potentially help reduce care fragmentation for patients.

### 6.2 Collaboration spillovers

In the present study, physicians' belonging to common insurance networks appeared to also be a large contributor to utility from patient collaboration and sharing. This finding is of particular interest as belonging to shared insurance networks for (non‐Medicare) market‐based plans was found to influence and shape physicians' patient sharing behavior of Medicare patients. Such spillovers may highlight the importance of relationship strength for physicians' patient sharing decisions, but it may also reflect some burden of initial referral—a physician can reduce the time impact (and overall cost) of sharing patients by sharing with the same colleagues repeatedly (this idea is supported by the literature surrounding team productivity and team familiarity, see Agha et al., 2018). 16This finding also bears some resemblance to the “norms hypothesis” (put forth by Newhouse & Marquis, 1978) in that physicians may share patients not on the basis of insurance of any given patient, but rather on the most likely insurance network requirements they encounter most frequently (across all of their patients—that is, Medicare and non‐medicare patients). Administrators seeking to improve care coordination or reduce leakage, as well as policy makers attempting to define ACOs, may consider the importance of examining the insurance network overlap of physicians within institutions or practice settings in order to capitalize on this potential driver of patient sharing.

### 6.3 Specialty/knowledge complementarity

The observed sorting on the basis of knowledge overlap is unexpected, especially considering that we are controlling for factors related to common institutional affiliation. It might be that physicians tend to form links with other physicians that are like them in terms of their skillsets due to homophily. This is supported by survey data reported by Meltzer et al. (2010), in which physicians tend to form social ties most frequently with those from the same specialty. In that case, the increased utility from patient sharing is reflective of this social bond between physicians. Alternatively, it might be a manifestation of defensive medicine where physicians collaborate with others that share their skillsets in order to reduce risks related to medical errors or misdiagnosis (see, e.g., Song, Sequist, & Barnett, 2014).

In either of these scenarios, it is possible that patient sharing despite knowledge overlap is indicative of inefficiency within the system and points to a potential source of care fragmentation. One solution may come from further adoption of the medical home model, in which the primary care physician serves as coordinator for all the specialists that an individual patient sees. However, it is also important to consider the role of specialist collaboration in diagnosis and treatment of complex or rare medical disorders—as Agha et al. (2018) point out, there is a trade‐off between the optimal strategy of the PCP seeking to reduce the effort of care coordination from referrals with quality derived from a patient having more options of specialists to see in order to find an ideal patient‐specialist match. Further research is needed to determine optimal network structure in cases where a large number of specialists are needed for effective care.

### 6.4 Study limitation and avenues for future work

The question that has motivated this study is: What drives the formation of physician patient sharing networks in Medicare? Importantly, this question asks for the channels that cause physician patient sharing. Although the present study attempts to formulate a structural micro‐founded model to address this question, and although it does control for a number of potential channels that may influence the formation of physician patient sharing ties, giving a causal interpretation to the estimated effects requires accepting a number of strong assumptions that are important to highlight.

First, identification within the present study rests on the assumption that links form independently conditional on physician‐pair observables (*HospitalAffiliation*_*ij*_,*X*_*ij*_) and, importantly, on the latent physician attributes (*A*_*i*_,*A*_*j*_). The ability to control for physician fixed effects is a strength of the method; however, it is possible that some of the findings may be driven by some unmeasured physician pair‐specific features that the study fails to account for. 17For additional discussion on issues related to endogeneity within matching models see, for example, Graham (2011).
18It is worth noting that the approach here will be able to capture some physician‐pair specific unobservables so long as these can be assumed to be additively separable, and thereby be picked up by the physician fixed effects. Reverse causality may furthermore be of concern in some instances. Given this limitation, the present study has put its main focus on exploring the channel related to shared hospital affiliation as these limitations may likely pose less of a threat to identification for institutional features of physicians then to other mechanisms such as, for example, insurance network effects. 19For example, in the case of insurance networks, while it appears probable that physician's sharing membership in common insurance networks may lead them to establish patient sharing ties, it is also a possibility that a patient sharing relationship can cause them to—over time—align in order to ensure they are part of the same insurance networks. This type of reverse causality argument, however, appears less probable in the case of shared hospital affiliation, as it is likely that sharing hospital affiliation may bring about a shared patient relation, but the reverse–two physicians who share patients decide to become affiliated with the same hospital—appears less likely.


Second, if we assume that the physician pair‐specific unobservables do not raise identification concerns, then a causal interpretation will here rest on assuming that the physician link specific utility function is accurately specified. The present study has followed the prior empirical literature on network formation and matching in adopting a simple and parsimonious model specification and empirical functional form. As noted by McCalman and Rysman (2019) and Fox and Bajari (2013), parsimony is an important model selection criterion within the early stages of this literature. Although parsimony may be an important guiding principle for selecting a model, imposing these restrictions may again call into question potential endogeneity concerns. A finding within the current study, however, that helps ameliorate some of these concerns is the result that the estimated model is able to satisfy a great majority of all the tetrad level inequalities implied by a pairwise stable equilibrium. This lends support in favor of the model's parsimonious specification.

The listed threats to identification of particular causal effects present opportunities for future work in this area. Although the present method accounts for latent physician specific unobservables, it does not estimate the physician fixed effects. Estimating these latent terms would open the possibility for conducting counterfactual analysis where researchers can begin to compare observed network structures, which may be pairwise stable, but not necessarily globally efficient, to alternative network configurations that can help improve efficiency. Additionally, extending the present analysis to also include non‐physician clinicians with whom physicians may also commonly share patients may present additional insights on patient sharing among health care professionals at large.

## 7 CONCLUSION

The present study finds that common hospital affiliation is a strong driver of patient sharing behavior. This study builds on a body of literature interested in understanding physicians' Medicare patient sharing behavior by implementing an empirical network formation model that allows one to measure the relative impact of a number of potential drivers of network formation while accounting for unobserved physician characteristics. Being able to control for unobservable influence due to, for example, physician reputation and quality is critical to establishing a credible case for causality. Understanding the drivers of physician patient sharing is of great policy relevance as many current health care market innovations focus on improving the way health care professionals collaborate in their care delivery.

## Supporting information



Manuscript_PhysicianNetworks_Revision2.pdf
