# Supplementary material for: The formation of physician patient sharing networks in medicare: Exploring the effect of hospital affiliation
Source: Health Econ. 2019 Oct 28;28(12):1435–48. doi: 10.1002/hec.3936 (PMC6899902; doi:10.1002/hec.3936)
Supplement: Supplementary file 1 — Manuscript_PhysicianNetworks_Revision2.pdf [file HEC-28-1435-s001.pdf]

The Formation of Physician Patient Sharing Networks in Medicare: Exploring the Effect of Hospital Affiliation

July 2019

Abstract

This study explores the forces that drive the formation of physician patient sharing networks. In particular, I examine the degree to which hospital affiliation drives physicians' sharing of Medicare patients. Using a revealed preference framework where observed network links are taken to be pairwise stable, I estimate the physicians' pair-specific values using a tetrad maximum score estimator that is robust to the presence of unobserved physician specific characteristics. I also control for a number of potentially confounding patient sharing channels, such as: (i) common physician group or hospital system affiliation, (ii) physician homophily, (iii) knowledge complementarity, (iv) patient side considerations related to both geographic proximity and insurance network participation, and (v) spillover from other collaborations. Focusing on the Chicago hospital referral region, I find that shared hospital affiliation accounts for 36.5% of the average pair-specific utility from a link. Implications for reducing care fragmentation are discussed. (*JEL:* C14, C31, D85, I11, I12, I13)

Keywords: Endogenous Network Formation, Homophily, Insurance Networks, Medicare, Physician Patient Sharing, Unobserved Degree Heterogeneity.

1 Introduction

Recent work has helped uncover important relationships between the underlying structure of physician patient sharing networks and overall healthcare outcomes (Barnett et al. (2012), Landon et al. (2018)). One aspect of network structure that is related to healthcare outcomes is care fragmentation. Fragmented service delivery occurs when a patient is seen by a large number of physicians, introducing risk for lapses in coordination of care, with growing evidence that network structures with large numbers of specialist providers (i.e. higher levels of fragmented care) are associated with higher healthcare utilization and higher costs (Agha et al. 2018, Agha, Frandsen, Rebitzer, 2019). However, despite the advances in connecting physician patient sharing networks with outcome measures, less is known about the factors that give rise to these physician networks in the first place. By combining knowledge of why physicians seek certain ties with what we know about the impact of these structures on outcomes, the hope is that stakeholders seeking to improve the current medical system will be able to develop strategies that provide appropriate physician incentives for achieving the desired patient sharing network structure.

One hurdle in accomplishing this aim has been that establishing a credible case for causality in the presence of unobservable physician specific characteristics that may play a role in physician patient sharing (e.g. unobserved reputation or quality measures) is particularly problematic in social network settings where the researcher commonly works with cross sectional data on a sparse network that exhibits considerable degree heterogeneity (see e.g. Chandrasekhar (2016) and Graham (2017)).[1] Since the sparsity of the network makes standard fixed effects

[1]A *sparse network* refers to a network where only a subset of all possible network links are present, while *degree heterogeneity* refers to the observation that some people within the network have several links, while others only have a few links. *Degree heterogeneity* is a function of both

approaches problematic within these settings, alternative methodologies have been developed for dealing with the identification issue introduced by unobserved degree heterogeneity (see e.g. Graham (2017) and Kim(2018)).[2] In the present study I utilize a revealed preference framework that assumes that the observed physician patient sharing network is the outcome of a strategic network formation game (a la Jackson and Wolinski (1996)), where the resulting network ties are assumed to be pairwise stable in that physicians do not want to deviate to either remove any existing ties or add any non-existing ties. The equilibrium notion of pairwise stability provides me with inequalities that are used to estimate the parameters of physicians' link specific utility (using a tetrad maximum score estimator), which allows me to account for physicians' degree heterogeneity that may stem from physician-specific unobservables.

Specifically, this study explores the degree to which physician patient sharing is driven by having a common hospital affiliation. Understanding the effect that a shared hospital affiliation has on physicians patient sharing behavior is of particular policy relevance as delineating accountable care organizations (ACOs) along the boundaries of hospital affiliated physicians has previously been proposed within the literature as a strategy for combating care fragmentation, and in turn, high costs and poor quality outcomes (Fisher et al., 2007). In order to attempt to capture a causal effect, I include a number of potentially confounding link formation channels in addition to controlling for physician-specific unobservables. The included control variables draw from trends noted by Landon et al. (2012) and Barnett et al. (2011) related to why physicians form collaboration relationships and share patients, including: 1) institutional affiliations beyond a

observable and *unobservable heterogeneities* in attributes across physicians.

[2] Pakes et al. (2015) and Pakes (2010) provide a general framework for dealing with these types of identification issues more broadly in the context of estimation with moment inequalities.

shared hospital affiliation; 2) physician homophily due to shared characteristics (e.g. gender and/or years of experience); 3) knowledge complementarity in terms of physician specialties; 4) patient considerations related to the geographic proximity and economic accessibility (based on insurance network inclusion) of other physicians; and 5) collaboration spillover, where physicians share patient groups due to existing collaboration ties and/or familiarity. Naturally, other physician idiosyncratic factors such as a physician's reputation for quality and service, his/her patient panel characteristics, and even personality, may factor into the number of patient sharing ties that one physician has when compared to another. As such, accounting for potential unobserved physician link heterogeneity is critical to evaluating the overall importance and relative significance of hospital affiliation on physicians' patient sharing.

The present study builds on, and contributes to, a number of different but related literatures. First, it contributes to the recent literature on empirical network formation estimation, where previous contributions have looked at airline networks (McCalman and Rysman (2019)) and social lending networks in India (Kim (2018)). The second is the literature on physician referrals, which has primarily focused on surveying physicians regarding their referral behavior (Barnett et al. (2011), Forrest et al. (2006), Gonzalez and Rizzo (1991), Kinchen et al. (2004)). While these studies provide important insights for factors that may contribute towards physicians' patient sharing, the present study differs by considering physician patient sharing (rather than direct referrals). Additionally, preferences for whom to collaborate with are elicited directly from shared patient data via a revealed preference approach rather than using survey data. The third related line of work has focused on using physician patient sharing data to approximate physician social/collaboration ties. Here, Barnett et al. (2011) and Landon

et al. (2012) provided the important validation for this methodology, while other studies have followed, establishing relationships between observed network structures and healthcare outcomes in terms of utilization, cost, and quality (see e.g. An et al. (2018), Barnett et al. (2012), and Pollack et al. (2012, 2014)) and to describe important properties of the physician patient sharing networks (Landon et al. (2012b)). The present study builds on, and contributes towards, this strand of work by focusing in on the physician incentives that give rise to the observed network structures studied within this literature, while paying close attention to the empirical issue of unobserved physician degree heterogeneity.

The dataset consists of 1,306 physicians (MD or DO) who practiced within the Chicago Hospital Referral Region in 2016 and who collectively had 12,091 patient sharing ties. The physician level data is unusually rich in that it spans information on physician: Medicare patient sharing, characteristics, affiliations (group, hospital and system), geographic practice location, and insurance plan participation across most individual (under 65), small-, mid-, and large-group market plans sold both on and off the federal and state exchanges as well as Medicare Advantage plans. Using this data I find that physician patient sharing appears strongly motivated by common hospital affiliation. Additionally, while control variables also appear to provide utility to physician patient sharing ties, the relative importance of each of these channels is found to vary considerably.

The rest of this paper is organized as follows: Section 2 outlines the basic model. Section 3 presents the empirical specification along with the tetrad maximum score estimator and the main variable definitions. Section 4 covers the data and descriptives. Section 5 reports the main results, Section 6 provides a discussion of the main findings, and Section 7 concludes.

2 Basic Model

Let $N = \{1, ..., n\}$ be the set of physicians within market m, and let i and j denote typical members of the N set. The collaboration (or mutual patient sharing status) of all the physicians in market m is given by the network (or adjacency matrix) $g = \{g_{i,j} \mid i, j \in N\}$, where elements are $g_{i,j} = 1$ if i and j are collaborators, and $g_{i,j} = 0$ if they are not.[3] Let the $g + g_{ij}$ denote network g with a link added between i and j, and $g - g_{ij}$ denote network g with a link removed between i and j. Define $N(i; g) = \{k \in N \mid g_{i,k} = 1\}$ as the set of agents with whom i has a mutual (two-way) collaboration (i.e. a link). The utility of a physician from a given network configuration g is written as the summed benefit from all of his/her collaboration ties. That is,

$$U_i(g) = \sum_{k \in N(i;g)}^{N} u_{ik}, \tag{1}$$

where u_{ij} is the benefit i receives from having j as a collaborator (i.e. this is a link specific payoff). The marginal benefit from i and j forming a link is given by:[4]

$$\begin{aligned} mu_{ij} &= (U_i(g + g_{ij}) + U_j(g + g_{ij})) - (U_i(g - g_{ij}) + U_j(g - g_{ij})) \\ &= \left(\sum_{k \in N(i;g+g_{ij})}^{N} u_{ik} + \sum_{k \in N(j;g+g_{ij})}^{N} u_{jk} \right) - \left(\sum_{k \in N(i;g-g_{ij})}^{N} u_{ik} + \sum_{k \in N(j;g-g_{ij})}^{N} u_{jk} \right) \\ &= (u_{ij} + u_{ji}) \\ &= 2u_{ij}, \end{aligned} \tag{2}$$

[3]As is standard, $g_{i,i} = 0$, $\forall\ i \in N$.

[4]Regarding the notation: if $g_{ij} = 1$, then $U_i(g+g_{ij}) = U_i(g)$, and if $g_{ij} = 0$, then $U_i(g-g_{ij}) = U_i(g)$. This captures the notion that a link can either exist or not. Also note that link specific payoffs are assumed to be symmetric in that $u_{ij} = u_{ji}$.

where $2u_{ij}$ captures the direct symmetric benefits that accrue to i and j from forming a direct link.

The equilibrium notion used is that of pairwise stability (due to Jackson and Wolinski (1996)). This states that the physician patient sharing network g is *pairwise stable* if:

(i) for every $g_{i,j} = 1$, $U_i(g) + U_j(g) \geq U_i(g - g_{i,j}) + U_j(g - g_{i,j}) \iff mu_{ij} \geq 0$; and

(ii) for every $g_{i,j} = 0$, $U_i(g) + U_j(g) \geq U_i(g + g_{i,j}) + U_j(g + g_{i,j}) \iff mu_{ij} \leq 0$.

Condition (i) states that if we observe a link between i and j ($g_{i,j} = 1$) then it must be the case that the overall payoff from having the link exceeds the payoff should i and j remove the link. Similarly, condition (ii) holds that if no link is observed between i and j ($g_{i,j} = 0$) then it must be the case that the joint payoff from not having a link is greater than that which would be obtained from i and j adding a link.

Next, we consider the empirical specification of the marginal utility and specify the tetrad inequalities that follow from our definition of pairwise stability and that will be used for the purpose of our parameter estimation.

3 Empirical Approach

3.1 Pair specific payoffs and tetrad inequality

In order to bring our model to data we need to specify the pair specific payoff term, mu_{ij}, and give it a functional form.[5] To this end, let this marginal utility

[5]Following prior work in this area I employ a parsimonious specification where the marginal utility has a additively separable functional form (see e.g. McCalman and Rysman (2019) in

be given by:

$$mu_{ij} = \gamma HospitalAffiliation_{ij} + X_{ij}^{'}\boldsymbol{\beta} + A_i + A_j + \xi_{ij}, \qquad (3)$$

where $HospitalAffiliation_{ij}$ captures i and $j's$ degree of shared hospital affiliation; X_{ij} denotes the other pair specific attributes that are observed in the data; A_i and A_j are the unobserved physician-level attributes (accounting for physician level degree heterogeneity), and ξ_{ij} is the pair-specific error term that is assumed to be independently and identically distributed across all physician pairs. Identification rests on the assumption that $E[\xi_{ij} \mid HospitalAffiliation_{ij}, X_{ij}, A_i, A_j] = 0$.

As has previously been noted (see e.g. Graham (2017) and Kim (2018)), the fact that the econometrician does not observe A_i or A_j poses an important threat to identification in the context of our problem, as omitting these physician fixed effects can result in biased estimates of our parameters $\theta = (\gamma, \boldsymbol{\beta})$.[6,7] Moreover, the sparsity of real world social networks make it problematic to estimate the unobserved fixed effects directly since most agents within these networks have few links (low degree). Alternative approaches for dealing with this issue have been suggested for inequality estimators within the matching (see e.g. Fox(2010)) and network (Kim (2018)) literatures.[8] This approach has us select a tetrad of

the case of a network formation application or Fox and Bajari (2013) in a matching setting).

[6]The issue stems from the fact that a given physician may have a lot of links due to some important unobserved characteristic–like for example, being a highly regarded physician due to having a reputation of providing high quality care, etc. This latent quality factor is hence captured by the A_i term.

[7]Potential threats to identification include unobserved pair-specific factors that are not additively separable, and therethrough not fully captured by the individual fixed effects. Further, while the approach does account for physician unobservables (A_i, A_j), our specification assumes these enter linearly and that they are not interacted with any of the pair-specific characteristics, X_{ij}. Additional details on study limitations are provided within the Discussion section (Section 6), and estimates from an alternative specification are included within the Online Appendix B as a robustness check.

[8]Graham (2017) uses a similar approach to deal with unobserved degree heterogeneity within his tetrad Logit model.

individuals–that is, four distinct individuals i, j, k and l, where $g_{ij} = 1$, $g_{kl} = 1$, $g_{il} = 0$, $g_{jk} = 0$, $g_{ik} = 0$, $g_{jl} = 0$. Here, pairwise stability implies that for the linked pairs ($g_{ij} = 1$, $g_{kl} = 1$) we have:

$$mu_{ij} \geq 0 \quad \wedge \quad mu_{kl} \geq 0. \tag{4}$$

While for the non-linked pairs we have (for $g_{il} = 0$, $g_{jk} = 0$):

$$mu_{il} \leq 0 \quad \wedge \quad mu_{jk} \leq 0, \tag{5}$$

and (for $g_{ik} = 0$, $g_{jl} = 0$):

$$mu_{ik} \leq 0 \quad \wedge \quad mu_{jl} \leq 0. \tag{6}$$

Combining inequalities (4) and (5) yields the tetrad inequality:

$$mu_{ij} + mu_{kl} \geq mu_{il} + mu_{jk}, \tag{7}$$

and similarly, combining (4) and (6) yields:

$$mu_{ij} + mu_{kl} \geq mu_{ik} + mu_{jl}. \tag{8}$$

From inequalities (7) and (8) it is clear that the individual level heterogeneity terms (A_i, A_j, A_k, A_l) will cancel out since they appear on both sides of the inequalities. As such, the omission of unobserved physician-level characteristics that may cause degree heterogeneity within the data will not bias our parameter estimates.

3.2 Variable Definitions

The physician pair specific variables ($HospitalAffiliation_{ij}$,X_{ij}) used within the empirical model control for a number of physician, patient, and institutional characteristics that have been identified within the literature as potential drivers of physicians' patient sharing. Here, I outline the variable definition for hospital affiliation and the controls for each of the other link formation channels.

Hospital Affiliation

To account for patient sharing that is motivated by shared hospital affiliation, I control for whether physicians i and j are members of a number of common hospitals. This is defined as: $cSameHospital_{ij} = |H_i \cap H_j|$, which returns a count for the number of overlapping hospitals between i and j (i.e. the cardinality of the intersection between the set of physician hospitals of i (H_i) and that of j).

Other Common Institutional Affiliations

It may also be the case that physicians within a particular group or system tend to share patients with other physicians within the boundaries of their shared organization. The measure for a shared physician practice is given by: $cSamePractice_{ij} = |PP_i \cap PP_j|$, where PP_i denotes the set of practices that i is affiliated with. Shared system affiliation is captured using: $dSameSystem = 1\,(|S_i \cap S_j| > 0)$, where S_i denotes the set of hospital systems that i is affiliated with, and $dSameSystem$ takes a value of 1 if i and j have at least one shared system and 0 otherwise.

Homophily

Patient sharing may occur along social or friendship ties that are the result of physician homophily–that is, where physicians form patient sharing ties on the

basis of their shared characteristics. To this end, I control for physician's gender and experience. I control for shared gender using: $Gender_{ij} = 1\left(G_i = G_j\right)$, where G_i denotes the gender of i and $1(.)$ denotes an indicator function that takes the value of 1 when i and j have the same gender. For experience, the squared difference of physician $i's$ and $j's$ years of experience captures potential assortative link formation on the basis of shared years of experience: $sExperience_{ij} = \left(E_i - E_j\right)^2$, where E_i denotes the years of experience of physician i.

Knowledge Complementarity

Patient sharing ties may be established on the basis of physicians' differences in their expertise, that is, there may be important knowledge complementarities of certain physicians that drive patient sharing. To capture physicians' overlap in terms of their knowledge I consider each physicians knowledge vector $K_i = \{s_{i1}, s_{i2}, ..., s_{in}\}$, where $s_{i1} = 1$ if physician i lists specialty s_{i1} as one of their specialties (and $s_{i1} = 0$ otherwise), and define i and $j's$ knowledge overlap as the uncentered correlation of K_i and K_j: $jSpecialty_{ij} = K_i K_j' / (\sqrt{K_i K_i'} + \sqrt{K_j K_j'})$. This measure takes values between 0 and 1, with 1 indicating perfectly overlapping specialties.

Patient Considerations

Patient sharing may be based on physicians' patient considerations. For example, distance may impose possible access to care issues for patients that the physician may wish to avoid if possible. To capture such considerations, I control for the distance between physician i and $j's$ practices using their zipcodes to compute the travel distance. That is, distance is defined as: $Distance_{ij} = d(Zipcode_i, Zipcode_j)$, where $d(.)$ measures the distance between the center of

$Zipcode_i$ and $Zipcode_j$. Other patient considerations may relate to the patient's insurance in the cases where the Medicare patients have Medicare Advantage coverage. The influence of shared insurance network affiliation is captured by: $cSameMA\_Network_{ij} = \left|N_i^{MA} \cap N_j^{MA}\right|$, where $N_i^{MA} = \{Plan_{i1}, Plan_{i2}, ..., Plan_{ip}\}$ is a set of all Medicare Advantage plans that physician i is part of.

Spillovers

Lastly, other professional interaction may lead physicians to share medicare patients. For example, it may be the case that physicians' sharing of other types of patient groups leads to the establishing of patient sharing ties that spillover and cause the physicians to also share Medicare patients. I investigate this possibility by controlling for physicians' shared participation in insurance networks for non-Medicare (i.e. "other") types of patients. This overlap is measured using: $cSameOther\_Network_{ij} = \left|N_i^{O} \cap N_j^{O}\right|$, where $N_i^{O} = \{Plan_{i1}^{O}, Plan_{i2}^{O}, ..., Plan_{ip}^{O}\}$ is a set of all non-Medicare Advantage plans that physician i is part of.

3.3 Tetrad Maximum Score Estimation

The estimator employed within this study is a tetrad-level maximum score estimator (a la Kim, 2018).[9] This estimator has important computational advantages that make it feasible to apply it to large scale networks like the physician patient sharing network within this study. Moreover, Kim (2018) shows that the utility parameters are point identified, and importantly that bias concerns related to individual-level unobserved heterogeneity is ameliorated by the fact that these individual fixed effects are canceled within the tetrad-level maximum score

[9]Early work on the maximum score estimator was done by Manski (1975), however, more recent work by Fox (2018, 2010), has applied the maximum score estimator toward the estimation of pairwise-stable two-sided matching problems.

estimator. The estimator in the case of one large market is given by:

$$Q(\beta) = \left(2\begin{pmatrix} N \\ 4 \end{pmatrix}\right)^{-1} \sum_{i,j,k,l\in T} \frac{1}{2}[1\left(s_{ijkl}(\theta) \geq s_{ikjl}(\theta)\right) + 1\left(s_{ijkl}(\theta) \geq s_{iljk}(\theta)\right)]. \tag{9}$$

In equation (9) we have $s_{ijkl}(\theta) = 1\left(X'_{ij}\theta + X'_{kl}\theta\right)((1-a_{ik})(1-a_{il})(1-a_{jk})(1-a_{jl}))$ where $a_{ik}, a_{il}, a_{jk}, a_{jl} \in A(g) = G + I$, with $A(g)$ denoting the adjusted adjacency matrix obtained by taking the actual adjacency matrix for network g (given by G) and adding an identity matrix to it so that the diagonal elements of $A(g)$ are equal to one rather than zero. The adjustment term $((1-a_{ik})(1-a_{il})(1-a_{jk})(1-a_{jl}))$ in $s_{ijkl}(\theta)$ ensures that the tetrads selected satisfy the requirements for pairwise stability and also help ensure the existence of a pairwise stable equilibrium as well as identification (see Jackson and Watts (2001, 2002) and Kim (2018)). In terms of identification, as is common for this type of estimators, we are able to identify relative magnitudes, but not an absolute level. As such, I impose a normalization where one of the θ parameters is set to ± 1 causing all the other parameters to be interpreted relative to this value (see e.g. Fox(2010)).

Estimation of the parameter vector (θ) is implemented using a differential evolution search algorithm, and confidence regions are computed using random subsampling (see e.g. Fox and Santiago (2014)).

4 Data and Descriptives

Focusing on the Chicago hospital referral region, I consider all physicians (with a MD or DO credentialing) that see Medicare patients and who are therefore present in the Centers for Medicare and Medicaid Services (CMS) Physician Compare registry in 2016. The Physician Compare dataset contains detailed physician level

information regarding physician characteristics such as gender, years of experience, schooling, primary and secondary specialties, along with institutional information regarding physician group and hospital affiliations. Group practice and hospital affiliations are determined using Medicare claims data. A physician is defined as affiliated with a given hospital if they provided services there to at least three different patients, on at least three different occasions, occurring within the last year. In line with previous work (see e.g. Barnett et al. (2012)), I remove physicians to whom referrals are less likely (i.e. physicians whose primary specialty is e.g. anesthesia, pathology, and radiology). The remaining physicians consist of 1,306 primary care physicians, medical and surgical specialists.[10] Additional physician level data on physicians' insurance network affiliations for both Medicare Advantage and other plans were sourced from the company Vericred. The non-Medicare Advantage plans span most individual (under 65), small-, mid-, and large-group market plans sold both on and off the federal and state exchanges.[11] Hospital system affiliation was linked from the American Hospital Association.

Lastly, the physician level data is linked with CMS's data on physicians' sharing of Medicare patients. The patient sharing network ties are constructed from Medicare Claims data and was provided by Carelink Labs. Here, the unipartite physician network is a simple projection of a bipartite physician-to-patient network (as described in, e.g., Barnett et al. (2012) or Landon et al. (2013)). Previous work has found that one would want a minimum of 9 shared patients for a network link to be considered a shared patient tie and to further reduce the risk

[10]For a complete list of physicians specialties, see the Online Appendix A.

[11]The insurance network data is for the 2016 plan period, while the Medicare Advantage network data is for 2017. While the use of 2017 Medicare Advantage Network data might introduce some noise into the data, I use plan CMS provided crosswalks in order to map the 2017 plans back to 2016 plans. In so doing I see that majority of the plans did not change between 2016 and 2017, with only about 3% of the plans being discontinued. These were excluded from the analysis.

of including spurious links that may be driven by patient choice (Barnett et al. (2011)). This notion of a minimum criterion for a link to denote a patient sharing tie is preserved within the data as per CMS privacy policies provider pairs who shared less than 11 distinct patients together (within the given time period) are not included in the data.

[TABLE 1]

Table 1 presents summary statistics at the physician-level. Here we see that physicians are on average affiliated with 1.9 hospitals, 1 practice, and 0.8 systems. Close to 30% of the physicians are female and the physicians have on average 27.9 years of experience and 1.5 specialties. Lastly, looking at the physicians insurance network memberships we see that they are on average part of 5.2 Medicare Advantage networks and 20.4 exchange (non Medicare-Advantage) networks.

[TABLE 2]

Next, Table 2 provides summary statistics for the pair-specific variables for three different cases: (i) the realized patient sharing ties within the data; (ii) the expected pair specific value from purely random link formation between any two physicians i and j within the data; and (iii) the expected pair values within a network where ties are formed randomly conditional on preserving the degree distribution of the realized patient sharing network.[12] Comparing the values across the realized values and those obtained across random link formation provides details on the extent to which observed links between physicians are assortative. A consistent trend is that the realized average link value is higher than either one of the values expected from random link formation. This observation lends

[12]The conditionally random network is constructed using the monte carlo sampling method put forth by Viger and Latapy (2005).

descriptive support toward physician collaboration ties being non-random and instead the outcome of physicians assortative (and disassortative) sorting into patient sharing relationships.

The finding of assortative link formation can further be illustrated by mapping out the physician patient sharing network and visually examining it for evidence of non-random network structures. This is done in Figure 1, where Panel A showcases the physician network for the Chicago hospital referral region (HRR). In Panel A we observe a number of clusters within the data, which again, suggests non-random link formations between physicians and considerable degree-heterogeneity among physicians. Panel B illustrates the network structure one would expect if physicians were to form links at random conditional on the observed degree distribution within the data. Comparing Panel B to that in Panel A we are further able to appreciate the amount of structure that exists within the observed physician network. Next, Panel C shows the actual network (as in Panel A), but highlights physicians with hospital affiliations at Northwestern Memorial Hospital (red), University of Chicago Medical Center (purple), Rush (yellow) and University of Illinois hospital (orange). Panel D similarly highlights four hospitals that make up Presence hospital system in Chicago. What is clear in both Panels C and D is that there appears to be clustering among physicians on the basis of hospital and hospital system affiliation. Panel E illustrates the set of physicians that are part of one of United Health's Medicare Advantage HMO plans within the data and highlight that this particular network has a significant existence within physicians affiliated with Presence hospital system and also among physicians associated with Northwestern Memorial, but has hardly any physicians affiliated with the University of Chicago in its network. Lastly, For the sake of comparison, Panel F highlights physicians associated with a PPO silver plan offered by the

Land of Lincoln Mutual Health Insurance Company on the exchange. This plan has a much wider coverage of physicians than the previously considered network.

[FIGURE 1]

The next section explores the relative effect of hospital affiliation on physicians' link specific utility by estimating the model put forth in Section 2.

5 Results

Table 3 presents the tetrad maximum score point estimates along with their 95% confidence regions (column 1), a relative contribution of each average effect compared to the average effect of *dGender* (column 2), and the relative contribution of each effect when compared to the absolute link specific utility (column 3).[13] The estimates were obtained from a random subsample from the set of tetrads (consisting of 57,834 tetrads and 115,668 inequalities). The 95% confidence regions were constructed by subsampling using 200 draws, each with a 30% subsample of physicians (see e.g. Fox and Santiago (2014) or Kim (2018)). Since the tetrad maximum score estimator is able to identify the relative, but not the absolute, magnitude of each marginal effect, I normalize the same gender dummy (*dGender*) to +1 and use it as the reference parameter.[14]

[TABLE 3]

The core hypothesis is that patient sharing will be driven by physicians having common hospital affiliations. I find support for this hypothesis as physi-

[13] The average absolute link utility is computed as: $\overline{u_{ij}} = \sum_i \left|\hat{\beta}_i\right| * \overline{x_i}$, where $\overline{x_i}$ denotes the average value of variable x_i, $|.|$ is the absolute value, and the sum is taken across all variables. Using this as the reference utility ensures that the percentage contributions of all effects sum up to 100%.

[14] The parameter for dGender was set to +1 as this yielded a higher score (with more inequalities being satisfied) than a -1 value did.

cians belonging to one or more common hospitals (*cSameHospital*) positively contribute towards physicians' link specific utility. Looking at column 1 (Estimate) in Table 3 we see that the marginal effect of shared hospital affiliation (*cSameHospital*) is 34 times that of shared gender (*dGender*).[15] To gain further insight about the relative contribution of physicians' shared hospital affiliation toward their link specific utility, we can look at columns 2 and 3 in Table 3. Column 2 reports the average contribution of shared hospital affiliation relative to the average contribution of shared gender. In this case, the figure is 46.7 ($\frac{\hat{\beta}_{cSameHospital} * \overline{cSameHospital}}{\hat{\beta}_{dGender} * \overline{dGender}} = \frac{34.063*0.896}{1*0.654} = 46.7$) which tells us that for the average physician patient sharing link, shared hospital affiliation contributes 46.7 times more towards the link utility than does physicians' shared gender. Column 3 reports the percentage contribution of physicians' shared hospital affiliation relative to the absolute link specific utility. Here, we note that shared hospital affiliation on average contributes 36.5% ($\frac{|\hat{\beta}_{cSameHospital}| * \overline{cSameHospital}}{\sum_i |\hat{\beta}_i| * \overline{x_i}} = \frac{34.063*0.896}{83.6196} = 0.365$) towards the absolute link specific utility.

Additional potential channels for patient sharing are further included as controls. Considerable variability in relative impact of these channels is noted. First, common physician practice groups (*cSamePractice*) also positively contribute to physician utility from link formation, as does belonging to a common hospital system (*dSameSystem*). Overall, motives due to these other shared institutional affiliations appear to contribute 24.5% of the absolute link specific utility.

Second, physician homophily is examined. Table 3 provides evidence of positive assortative link formation among physicians with a common gender (*dSameGender*), and years of experience (*sExperience*). Overall, homophily arguments are found

[15] This relative effect is computed by dividing the same hospital estimate by the same gender estimate, that is: $\frac{\hat{\beta}_{cSameHospital}}{\hat{\beta}_{dGender}} = \frac{34.063}{1} = 34.063$.

to contribute a total of 1.9% of the absolute link specific utility.

Third, it was stated that physicians may form patient sharing ties on the basis of the complementarity of their knowledge. Table 3 shows that we in fact find the opposite result–physicians tend to form links with other physicians who share some degree of knowledge overlap with them (*jSpecialty*), and the benefit they derive from such a link appears to be increasing in the degree to which their skill sets overlap. In total, I find that link formation driven by knowledge overlap contributes 3.2% of the absolute link specific utility.

Fourth, physicians' patient considerations can influence who they share patients with. Specifically, distance (*Distance*) between two physicians offices negatively affects their link specific utility. An additional supporting finding is that physicians' link utility increases with the number of Medicare Advantage insurance plans ($cSameMA\_Networks$) that they have in common. The complete contribution is here 11.6% of the absolute link specific utility.

Finally, sharing of a particular patient group was influenced by physicians' collaboration on other patient groups. This spillover effect was controlled for using the count for all other (non-Medicare Advantage) insurance plans that the physicians had in common ($cSameOther\_Networks$). The link specific utility is increasing in the number of insurance networks that the physicians share, and overall, I find that the contribution due to this spillover effect contributes 22.3% towards the absolute link specific utility.

6 Discussion

In the present study, I apply a revealed preference framework to estimate physicians' link specific utility, using a rich dataset that allows me to focus on the

effect of physicians' common hospital affiliation on patient sharing and to control for a number of other potentially confounding link formation channels, while also accounting for physician degree heterogeneity from latent physician characteristics. To summarize, I find considerable variation in the relative contribution of each channel towards the absolute link specific utility, where shared hospital affiliation contributes the most, followed by other institutional affiliations, collaboration spillovers, patient considerations, specialty/knowledge complementarity and homophily. In what follows I seek to discuss some of the implications of these results for administrators and policy makers and discuss limitations of the current study with potential for future work.

Institutional Affiliation

The central finding of this paper is that shared hospital affiliation yields most of the link specific utility for physicians. This suggests that expanding physician and physician practice alignment with hospitals/hospital systems may be a potential strategy for reducing the fragmentation of patient care beyond current institutional boundaries (conditional on the number of links being the same). It is interesting to note that in the last couple of years we have in fact observed such a trend with an increased number of physician practices aligning themselves with hospitals/hospital systems (See e.g. Kane (2019)). From a patient perspective, it may suggest that selecting a physician within one affiliation network increases the likelihood of referral to a peer within the same practice, hospital, or health care system.

For the administrator, the finding that hospital affiliation plays a key role in physician patient sharing carries implications for minimizing patient "leakage," which occurs when a patient leaves one institutional system to receive care from

another. Managing and reducing patient leakage beyond the boundaries of physicians' core care team is of importance to both the reduction of patient care fragmentation, but also to institutional bottom lines. The results found here suggest that physician group alignment with a hospital or institutional system may increase within system patient sharing, resulting in a move towards within system collaboration that occurs organically. These findings are consistent with those reported by Carlin et al. (2016) and Walden (2016), where the authors demonstrate that physicians under common ownership begin to refer to one another following a hospital merger.

From a policy point of view, the results reported here can be beneficial in the move towards defining Accountable Care Organizations. Prior work has suggested that using physician patient sharing data to select ACOs can possibly lead to better adoption by tapping into already existing network infrastructure among physicians (Landon et al. (2013)). The finding that shared hospital affiliation contributes the most towards physician's link specific utility suggests that forming ACOs around the institutional boundaries of hospitals can potentially help reduce care fragmentation for patients.

Collaboration Spillovers

In the present study, physicians' belonging to common insurance networks appeared to also be a large contributor to utility from patient collaboration and sharing. This finding is of particular interest as belonging to shared insurance networks for (non-Medicare) market based plans was found to influence and shape physicians' patient sharing behavior of Medicare patients. Such spillovers may highlight the importance of relationship strength for physicians' patient sharing decisions, but it may also reflect some burden of initial referral—a physician can

reduce the time impact (and overall cost) of sharing patients by sharing with the same colleagues repeatedly (this idea is supported by the literature surrounding team productivity and team familiarity, see Agha et al 2018).[16] Administrators seeking to improve care coordination or reduce leakage, as well as policy makers attempting to define ACOs, may consider the importance of examining the insurance network overlap of physicians within institutions or practice settings in order to capitalize on this potential driver of patient sharing.

Specialty/Knowledge Complementarity

The observed sorting on the basis of knowledge overlap is unexpected, especially considering that we are controlling for factors related to common institutional affiliation. It might be that physicians tend to form links with other physicians that are like them in terms of their skillsets due to homophily. This is supported by survey data reported by Meltzer et al. (2010), in which physicians tend to form social ties most frequently with those from the same specialty. In that case, the increased utility from patient sharing is reflective of this social bond between physicians. Alternatively, it might be a manifestation of defensive medicine where physicians collaborate with others that share their skillsets in order to reduce risks related to medical errors or misdiagnosis (see e.g. Song et al. (2014)).

In either of these scenarios, it is possible that patient sharing despite knowledge overlap is indicative of inefficiency within the system and points to a potential source of care fragmentation. One solution may come from further adoption of the medical home model, in which the primary care physician serves as coordinator for all the specialists that an individual patient sees. However, it is also important to

[16]This finding also bares some resemblance to the 'norms hypothesis' (put forth by Newhouse and Marquis (1987)) in that physicians may share patients not on the basis of insurance of any given patient, but rather on the most likely insurance network requirements they encounter most frequently (across all of their patients–i.e. Medicare and non-medicare patients).

consider the role of specialist collaboration in diagnosis and treatment of complex or rare medical disorders—as Agha and colleagues (2018) point out, there is a trade-off between the optimal strategy of the PCP seeking to reduce the effort of care coordination from referrals with quality derived from a patient having more options of specialists to see in order to find an ideal patient-specialist match. Further research is needed to determine optimal network structure in cases where a large number of specialists are needed for effective care.

Study Limitation and Avenues for Future Work

The question that has motivated this study is: what drives the formation of physician patient sharing networks in Medicare? Importantly, this question asks for the channels that cause physician patient sharing. While the present study attempts to formulate a structural micro-founded model to address this question, and while it does control for a number of potential channels that may influence the formation of physician patient sharing ties, giving a causal interpretation to the estimated effects requires accepting a number of strong assumptions that are important to highlight.

First, identification within the present study rests on the assumption that links form independently conditional on physician-pair observables $(HospitalAffiliation_{ij}, X_{ij})$ and, importantly, on the latent physician attributes (A_i, A_j). The ability to control for physician fixed effects is a strength of the method, however, it is possible that some of the findings may be driven by some unmeasured physician pair-specific features that the study fails to account for.[17,18] Reverse causality may

[17]For additional discussion on issues related to endogeneity within matching models see e.g. Graham (2011).

[18]It is worth noting that the approach here will be able to capture some physician-pair specific unobservables so long as these can be assumed to be additively separable, and thereby be picked up by the physician fixed effects.

furthermore be of concern in some instances. Given this limitation, the present study has put its main focus on exploring the channel related to shared hospital affiliation as these limitations may likely pose less of a threat to identification for institutional features of physicians then to other mechanisms such as, e.g., insurance network effects.[19]

Second, if we assume that the physician pair-specific unobservables do not raise identification concerns, then a causal interpretation will here rest on assuming that the physician link specific utility function is accurately specified. The present study has followed the prior empirical literature on network formation and matching in adopting a simple and parsimonious model specification and empirical functional form. As noted by McCalman and Rysman (2019) and Fox and Bajari (2013), parsimony is an important model selection criterion within the early stages of this literature. While parsimony may be an important guiding principle for selecting a model, imposing these restrictions may again call into question potential endogeneity concerns. A finding within the current study, however, that helps ameliorate some of these concerns is the result that the estimated model is able to satisfy a great majority of all the tetrad level inequalities implied by a pair-wise stable equilibrium. This lends support in favor of the model's parsimonious specification.

The listed threats to identification of particular causal effects present opportunities for future work in this area. While the present method accounts for latent physician specific unobservables, it does not estimate the physician fixed

[19]For example, in the case of insurance networks, while it appears probable that physician's sharing membership in common insurance networks may lead them to establish patient sharing ties, it is also a possibility that a patient sharing relationship can cause them to–over time–align in order to ensure they are part of the same insurance networks. This type of reverse causality argument, however, appears less probable in the case of shared hospital affiliation, as it is likely that sharing hospital affiliation may bring about a shared patient relation, but the reverse–two physicians who share patients decide to become affiliated with the same hospital–appears less likely.

effects. Estimating these latent terms would open the possibility for conducting counterfactual analysis where researchers can begin to compare observed network structures, which may be pairwise stable, but not necessarily globally efficient, to alternative network configurations that can help improve efficiency. Additionally, extending the present analysis to also include non-physician clinicians with whom physicians may also commonly share patients may present additional insights on patient sharing among healthcare professionals at large.

7 Conclusion

The present study finds that common hospital affiliation is a strong driver of patient sharing behavior. This study builds on a body of literature interested in understanding physicians' Medicare patient sharing behavior by implementing an empirical network formation model that allows one to measure the relative impact of a number of potential drivers of network formation while accounting for unobserved physician characteristics. Being able to control for unobservable influence due to, for example, physician reputation and quality is critical to establishing a credible case for causality. Understanding the drivers of physician patient sharing is of great policy relevance as many current healthcare market innovations focus on improving the way healthcare professionals collaborate in their care delivery.

References

Agha, L., B. Frandsen, and J. B. Rebitzer, 2019, “Fragmented division of labor and healthcare costs: Evidence from moves across regions â”. *Journal of Public Economics*, vol. 169, pp. 144–159.

Agha, L., K. Marzilli Ericson, et al., 2018, “Team Formation and Performance: Evidence from Healthcare Referral Networks”. *Mimeo*, pp. 1–39.

An, C. et al., 2018, “Analysis of the U.S. patient referral network”. *Statistics in Medicine*, vol. 37, no. 5, pp. 847–866.

Barnett, M. L., N. A. Christakis, et al., 2012, “Physician Patient-sharing Networks and the Cost and Intensity of Care in US Hospitals”. *Medical Care*, vol. 50, no. 2, pp. 152–160.

Barnett, M. L., B. E. Landon, et al., 2011, “Mapping physician networks with self-reported and administrative data”. *Health Services Research*, vol. 46, no. 5, pp. 1592–1609.

Barnett, M. L., Z. Song, and B. E. Landon, 2012, “Trends in physician referrals in the United States, 1999-2009”. *Archives of Internal Medicine*, vol. 172, no. 2, pp. 163–170.

Carlin, C. S., R. Feldman, and B. Dowd, 2016, “The Impact of Hospital Acquisition of Physician Practices on Referral Patterns”. *Health Economics*, vol. 25, pp. 439–454.

Chandrasekhar, A. G., 2016, “Econometrics of Network Formation”. *Handbook of the Economics of Networks*, pp. 303–357.

Fisher, Elliott S et al., 2007, “Creating Accountable Care Organizations: The Extended Hospital Medical Staff: A new approach to organizing care and ensuring accountability.” *Health Affairs*, vol. 26, no. 1, pp. 44–57.

Forrest, C. B. et al., 2006, “Primary care physician specialty referral decision making: Patient, physician, and health care system determinants”. *Medical Decision Making*, vol. 26, no. 1, pp. 76–85.

Fox, J., 2010, “Identification in Matching Games”. *Quantitative Economics*, vol. 1, no. 2, pp. 203–254.

— , 2018, “Estimating Matching Games with Transfers”. *Quantitative Economics*, vol. 9, pp. 1–38.

Fox, J. and D. Santiago, 2014, “A Toolkit for Matching Maximum Score Estimation and Point and Set Identified Subsampling Inference”.

Franks, P. et al., n.d., “Variations in primary care physician referral rates”. *Health Services Research*, no. 1, pp. 323–329.

Gonzalez, Martin L. and John A. Rizzo, 1991, “Physician referrals and the medical market place”. *Medical Care*, vol. 29, no. 10, pp. 1017–1027.

Graham, B. S., 2011, “Econometric Methods for the Analysis of Assignment Problems in the Presence of Complementarity and Social Spillovers”. *Handbook of Social Economics*, vol. 1, no. 1B, pp. 965–1052.

— , 2017, “An Econometric Model of Network Formation With Degree Heterogeneity”. *Econometrica*, vol. 85, no. 4, pp. 1033–1063.

Jackson, M. O. and A. Watts, 2002, “On the formation of interaction networks in social coordination games”. *Games and Economic Behavior*, vol. 41, no. 2, pp. 265–291.

Jackson, M. O. and A. Wolinsky, 1996, “A Strategic Model of Social and Economic Networks”. *Journal of Economic Theory*, vol. 71, pp. 44–74.

Jacobson, G. et al., 2017, *Medicare Advantage 2017 Spotlight: Enrollment Market Update*. Tech. rep. Kaiser Family Foundation.

Kane, C. K., 2018, "2018 Benchmark Survey: For the First Time, Fewer Physicians are Owners Than Employees". *American Medical Association - Policy Research Perspectives*, pp. 1–16.

Kim, J. S., 2018, "Structural Estimation of Pairwise Stable Networks: An Application to Social Networks in Rural India". *Mimeo*, pp. 1–35.

Kinchen, K. S. et al., 2004, "Referral of patients to specialists: Factors affecting choice of specialist by primary care physicians". *Annals of Family Medicine*, vol. 2, no. 3, pp. 245–252.

Landon, B. E., N. L. Keating, M. L. Barnett, et al., 2012, "Variation in Patient-Sharing Networks of Physicians Across the United States". *JAMA*, vol. 308, no. 3, pp. 265–273.

Landon, B. E., N. L. Keating, J. P. Onnela, et al., 2018, "Patient-sharing networks of physicians and health care utilization and spending among medicare beneficiaries". *JAMA Internal Medicine*, vol. 178, no. 1, pp. 66–73.

Landon, B. E., J. P. Onnela, et al., 2013, "Using Administrative Data to Identify Naturally Occurring Networks of Physicians". *Medical Care*, vol. 51, no. 8, pp. 715–721.

Manski, C. F., 1975, "Maximum Score Estimation of the Stochastic Utility Model of Choice". *Journal of Econometrics*, vol. 3, pp. 265–291.

McCalman, P. and M. Rysman, 2016, "Arline Services Agreements: A Structural Model of Network Formation". *Mimeo*, pp. 1–31.

Meltzer, D. et al., 2010, "Exploring the use of social network methods in designing healthcare quality improvement teams". *Social Science and Medicine*.

Newhouse, J. P. and S. Marquis, 1978, "The Norms Hypothesis and the Demand for Medical Care". *The Journal of Human Resources*, vol. 13, no. 1978, pp. 159–182.

Pakes, A., 2010, "Alternative Models for Moment Inequalities". *Econometrica*, vol. 78, no. 6, pp. 1783–1822.

Pakes, A. et al., 2015, "Moment Inequalities and Their Application". *Econometrica*, vol. 83, no. 1, pp. 315–334.

Pollack, C. E., H. Wang, et al., 2014, "Physician Social Networks and Variation in Rates of Complications After Radical Prostatectomy". *Value in Health*, vol. 17, no. 5, pp. 611–618.

Pollack, C. E., G. Weissman, et al., 2012, "Physician social networks and variation in prostate cancer treatment in three cities". *Health Services Research*, vol. 47, no. 1 PART 2, pp. 380–403.

Song, Z., T. D. Sequist, and M. L. Barnett, 2014, "Patient referrals: A linchpin for increasing the value of care". *JAMA*, vol. 312, no. 6, pp. 597–598.

Viger, F. and M. Latapy, 2005, "Fast generation of random connected graphs with prescribed degrees". *CoRR*, vol. abs/cs/0502085, pp. 1–14.

Walden, E., 2016, "Can Hospitals Buy Referrals? The Impact of Physician Group Acquisitions on Market-Wide Referral Patterns". *Mimeo.*

Watts, A. and M. O. Jackson, 2001, "The existence of pairwise stable networks". *Seoul Journal of Economics*, vol. 14, no. 3, pp. 299–321.

Tables and Figures

Variable	Mean	Std. Dev.	N
cHospital	1.855	1.264	1,306
cPractice	1.048	0.560	1,306
cSystem	0.834	0.594	1,306
dFemale	0.298	0.457	1,306
cExperience_yrs	27.93	10.046	1,306
cSpecialties	1.467	0.636	1,306
cMA_Networks	5.172	5.852	1,306
cOther_Networks	20.382	8.199	1,306

Table 1: Summary Statistics at Physician-Level. Prefix: "d" denotes a dummy variable; and "c" a count variable measure.

	(i) Realized Links			***(ii) Random Links***			***(iii) Random Links | degree dist.***		
**Variable**	***Mean***	**SD**	**N**	***Mean***	**SD**	**N**	***Mean***	**SD**	**N**
cSameHospital	*0.896*	0.604	12091	*0.108*	0.335	853471	*0.148*	0.4	12,091
cSamePractice	*0.521*	0.63	12091	*0.06*	0.266	853471	*0.078*	0.302	12,091
dSameSystem	*0.595*	0.491	12091	*0.092*	0.289	853471	*0.123*	0.328	12,091
dSameGender	*0.654*	0.476	12091	*0.582*	0.493	853471	*0.63*	0.483	12,091
sExperience	*190.290*	253.587	12091	*202.171*	264.447	853471	*203.608*	264.138	12,091
jSpecialty	*0.262*	0.355	12091	*0.145*	0.294	853471	*0.211*	0.317	12,091
Distance_KM	*5.286*	8.403	12086	*11.302*	8.411	852165	*11.946*	8.861	12,086
cSameMA_Networks	*1.843*	3.046	12091	*1.324*	2.658	853471	*1.269*	2.437	12,091
cSameOther_Networks	*15.127*	6.689	12091	*12.804*	6.788	853471	*13.721*	6.123	12,091

Table 2: Summary Statistics for Physician-Pair Variables. Prefix: "d" a dummy variable; "c" a count variable; "j" an uncentered correlation; and "s" a squared difference measure. Part: (i) provides results from the patient sharing data; Part (ii) provides the expected results in the case of random link formation; and Part (iii) lists the results one would expect if link formation was random conditional on preserving the degree distribution within the observed data.

		(1)	(2)	(3)
**Link Channel**	**Variable**	**Estimate**	**Relative Contribution**	**% Absolute Contribution**
Hospital Affiliations	cSameHospital	34.063<br>(11.612, 43.192)	46.7	36.5%
Other Affiliations	cSamePractice	31.613<br>(4.883 , 39.379)	25.2	19.7%
	dSameSystem	6.728<br>(5.558, 8.7481)	5.1	4.8%
Homophily	dGender	1<br>(-,-)	1	0.8%
	sExperience	-0.005<br>(-0.008 , 0.004)	-1.5	1.1%
Specialty	jSpecialty	10.279<br>(7.583 , 13.751)	4.1	3.2%
Patient Considerations	cSameMA_Networks	0.499<br>(0.417, 0.694)	1.4	1.1%
	Distance	-1.653<br>(-2.180, -1.466)	-13.4	10.5%
Spillovers	cSameOther_Networks	1.231<br>(1.058, 1.655)	28.5	22.3%
	Numb. Links	12,086	-	-
	Numb. Inequalities	115,668	-	-
	% Ineq. Satisfied	98.478	-	-

Table 3: Maximum Score Estimates: A random sample 115,668 of all inequalities was used. 95% confidence regions were constructed using 200 random draws of a 30% subsample of physicians. The same gender dummy (*dGender*) is normalized to +1 and it is used as the reference parameter estimate column. The average link utility effect relative to that of the average same gender effect is reported in the column (Relative Contribution). For example, the relative effect of *cSameHospital* is given by: $\frac{\hat{\beta}_{cSameHospital}*\overline{cSameHospital}}{\hat{\beta}_{dGender}*\overline{dGender}} = \frac{34.063*0.896}{1*0.654} = 46.7$. The percentage contribution (% Absolute Contribution) of each effect is given by its relative absolute contribution towards the link specific utility obtained by multiplying the estimated parameter by the corresponding variable evaluated at its average population value, and dividing this by the overall link utility. For example, the percentage contribution of *cSameHospital* is given by: $\frac{\left|\hat{\beta}_{cSameHospital}\right|*\overline{cSameHospital}}{\sum_i \left|\hat{\beta}_i\right|*\overline{x_i}} = \frac{34.063*0.896}{83.6196} = 0.365$, where $\overline{x_i}$ denotes the average value of variable x_i.

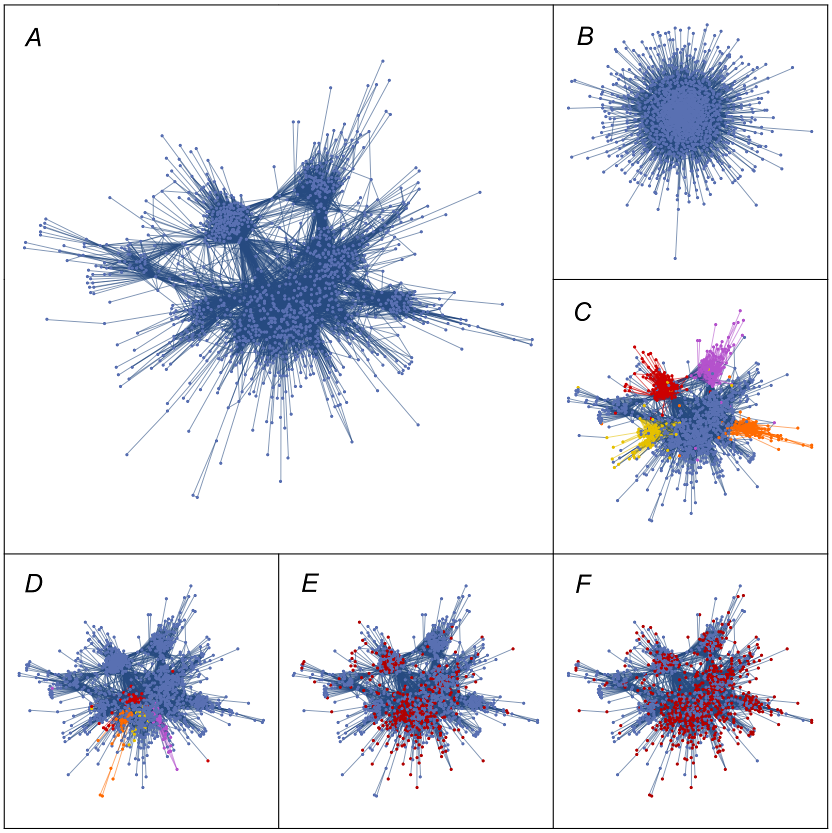

Figure 1: Panel A: Physician network (Chicago HRR); Panel B: Random network with the same degree distribution as the physician network; Panel C: 4 hospitals highlighted; Panel D: hospital system highlighted; Panel E: Medicare Advantage insurance network highlighted; and Panel F: Market based insurance plan highlighted.

Online Appendix

Appendix A: Additional Data Descriptives

Appendix A provides a number of supplemental data descriptives beyond those presented within the main text. Firstly, it presents additional figures related to the physician patient sharing networks that help highlight the amount of structure and also the level of sparsity and degree heterogeneity that is present within the data. Second, correlation tables at both the physician and physician-pair levels are presented for key variables. Third, frequency counts for variables related to physician specialty and affiliations (at the practice, hospital and system level) are provided along with details on physician reported areas of primary specialty. Lastly, descriptives are provided for variables at the tetrad inequality level which provide additional support–beyond that shown within Table 2–for physician sorting (both assortative and disassortative) on key physician-pair level characteristics.

Network Structure

Figure 1 within the main text contrasts the physician patient sharing network that we observe within the data (Panel A in Figure 1) with the network structure we would expect to observe if links were formed at random, conditional on preserving the degree distribution of the the realized data (panel B in Figure 1). Figure 2 provides an additional view of the realized network when compared to the random network along with each of the networks corresponding adjacency matrix plots. In the top right quadrant of Figure 2, we have a visualization of the realized network structure within the adjacency matrix, while in the bottom right quadrant we have the same visualization for the random network (which preserves

the network structure of the actual data). The adjacency matrix plots help reinforce what one already sees from the network structures themselves, which is that the realized network data contains evidence of considerable structure, something that the random graph does not. This is suggestive of physician patient sharing patterns being formed in a non-random fashion, which is further supported by the descriptive statistics reported within Table 2 (of the main text).

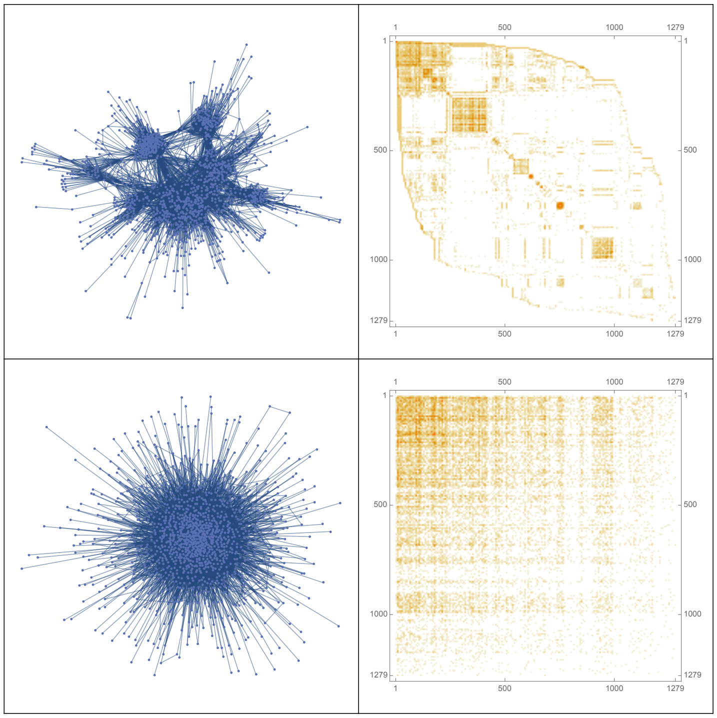

Figure 2: Realized Network Vs. Random Network

The degree distribution of the physician patient sharing data is provided within Figure 3. The long right-tail highlights the level of degree heterogeneity within the network. Degree heterogeneity and sparsity are common features of real world social networks, and as was noted within the main text, these features of the network impose challenges for traditional fixed effect approaches as many individuals have a low number of connections within the data.

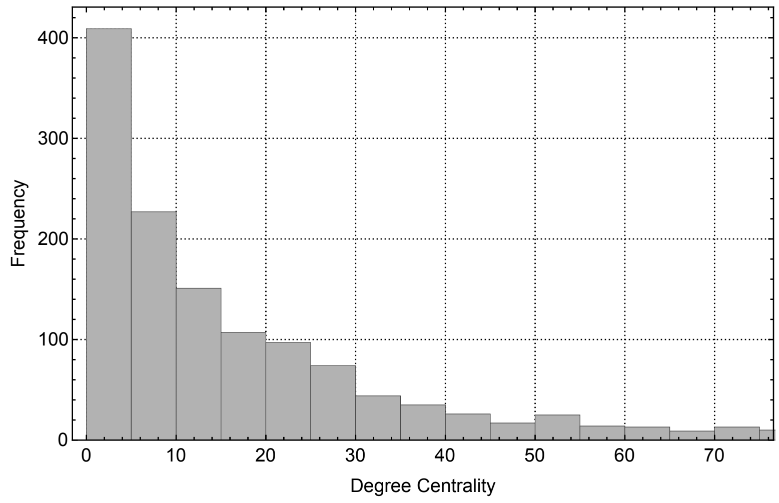

Figure 3: Physician Degree Distribution

Data Descriptives

Correlation Tables

Table 4 provides the correlation between individual physician level variables. These variables are importantly physician specific, as opposed to physician-pair specific.

Variables	dFemale	Experience	cSpecialties	cPractice	cHospitals	cSystem	cMA_Nets	cOther_Nets
dFemale	1.000							
Experience_yrs	-0.229	1.000						
cSpecialties	-0.076	0.039	1.000					
cPracticeGroup_Affiliations	-0.008	-0.111	0.072	1.000				
cHospital_Affiliations	-0.110	-0.028	0.045	-0.027	1.000			
cSystem_Affiliations	0.005	-0.037	-0.028	0.134	0.321	1.000		
cMA_Networks	-0.062	0.054	0.017	0.086	0.120	-0.016	1.000	
cOther_Networks	-0.064	0.040	0.007	0.036	0.199	-0.050	0.262	1.000

Table 4: Cross-Correlation Table for Physician-Level Variables. Prefix: "d" denotes a dummy variable; and "c" a count variable measure.

Table 5, on the other hand, provides the correlation between physician-pair level variables.

Variables	dGender	sExperience	jSpecilty	cSamePrac	cSameHosp	cSameSys	Distance	cMA_Nets	cOther_Nets
dSameGender	1.000								
sExperience	0.030	1.000							
jSpecialty	-0.021	0.021	1.000						
cSamePratice	-0.135	-0.031	0.053	1.000					
cSameHospital	-0.013	-0.049	-0.033	0.189	1.000				
cSameSystem	-0.056	-0.047	-0.070	0.343	0.467	1.000			
Distance	0.041	0.056	0.025	-0.319	-0.209	-0.292	1.000		
cSameMA_Nets	0.020	0.020	0.035	0.002	0.090	0.025	-0.014	1.000	
cSameOther_Nets	0.025	-0.003	0.055	-0.043	0.150	-0.057	0.014	0.151	1.000

Table 5: Cross-Correlation Table for Physician-Pair Variables. Prefix: "s" denotes the squared difference; "d" a dummy variable; "c" a count variable; and "j" an uncentered correlation measure.

Frequency Counts

Tables 6 and 7 provide frequency counts for some of the main variables along with a listing of the primary specialties of physicians within the dataset. Beginning with Table 6, we see that 58% of the physician sample report one specialty, while the remaining 42% of physicians report two or more specialties. Looking at the number of practice count frequencies, we note that 90% of the sample have two or fewer group practice affiliations. In terms of hospital affiliations we see that while a majority of physicians hold only one affiliation (47%), a considerable number (48%) hold two or more affiliations. Lastly, the hospital system affiliation count ranges from zero to three in frequency with 66% of physicians belonging to one system.

	Specialty Count		*Practice Count*		*Hospital Count*		*System Count*	
**Count**	Number	Percent	Number	Percent	Number	Percent	Number	Percent
**0**	0	0	160	12	61	5	346	26
**1**	781	60	936	72	622	48	842	64
**2**	455	35	199	15	323	25	107	8
**3**	56	4	9	1	135	10	11	1
**4**	13	1	2	0	75	6	0	0
**5**	1	0	0	0	90	7	0	0
**Total**	1,306	100	1,306	100	1,306	100	1,306	100

Table 6: Frequency Table For Physician's Specialty Count and Affiliations. Note that for each of the count variables, the "Number" column reports the number of physicians (out of the total 1,306 physicians within the sample) that are present within each bin, while the "Percent" reports the corresponding percentage that that number of physicians make up of the whole (1,306 physician) sample.

Next, Table 7 provides details on the primary specialties of the physician sample. In total we have here 55 different primary specialties, with the top three consisting of: Internal Medicine; Cardiovascular Disease (Cardiology); and Family Practice.

Item	Number	Percent
ALLERGY/IMMUNOLOGY	3	0
CARDIAC ELECTROPHYSIOLOGY	11	1
CARDIAC SURGERY	5	0
CARDIOVASCULAR DISEASE (CARDIOLOGY)	78	6
COLORECTAL SURGERY (PROCTOLOGY)	4	0
CRITICAL CARE (INTENSIVISTS)	8	1
DERMATOLOGY	23	2
ENDOCRINOLOGY	17	1
FAMILY PRACTICE	102	8
GASTROENTEROLOGY	50	4
GENERAL PRACTICE	9	1
GENERAL SURGERY	34	3
GERIATRIC MEDICINE	9	1
GERIATRIC PSYCHIATRY	1	0
GYNECOLOGICAL ONCOLOGY	5	0
HAND SURGERY	4	0
HEMATOLOGY	7	1
HEMATOLOGY/ONCOLOGY	60	5
HOSPICE/PALLIATIVE CARE	1	0
HOSPITALIST	10	1
INFECTIOUS DISEASE	26	2
INTERNAL MEDICINE	275	21
INTERVENTIONAL CARDIOLOGY	11	1
MEDICAL ONCOLOGY	15	1
NEPHROLOGY	59	5
NEUROLOGY	47	4
NEUROSURGERY	14	1
NUCLEAR MEDICINE	6	0
OBSTETRICS/GYNECOLOGY	37	3
OPHTHALMOLOGY	72	6
ORTHOPEDIC SURGERY	58	4
OTOLARYNGOLOGY	24	2
PAIN MANAGEMENT	6	0
PEDIATRIC MEDICINE	3	0
PERIPHERAL VASCULAR DISEASE	1	0
PHYSICAL MEDICINE AND REHABILITATION	25	2
PLASTIC AND RECONSTRUCTIVE SURGERY	6	0
PSYCHIATRY	27	2
PULMONARY DISEASE	54	4
RADIATION ONCOLOGY	25	2
RHEUMATOLOGY	19	1
SLEEP MEDICINE	1	0
SPORTS MEDICINE	5	0
SURGICAL ONCOLOGY	8	1
THORACIC SURGERY	8	1
UROLOGY	26	2
VASCULAR SURGERY	7	1
Total	1,306	100

Table 7: Frequency Table For Physician's Primary Specialty.

Assortative (and Disassortative) Sorting

Equations (7) and (8) within the main text capture the tetrad level inequalities. Focusing on Equation (7) for tetrad (i, j, k, l), where ij are connected and kl are connected, we have that $mu_{ij} + mu_{kl} \geq mu_{il} + mu_{jk}$ is implied by pairwise stability, which in turn suggests that physicians sort (assortatively and dissasortatively) into physician patient sharing relationships across pair-specific observables and individual specific unobservables. The plausibility of sorting into physician pairs on the basis of observables is explored within Table 2 of the main text, however, an additional check can be done by looking at whether $X_{ij} + X_{kl} - (X_{il} + X_{jk}) \lesseqqgtr 0$ holds. Here, a positive value is suggestive of positive assortative sorting on the physician-pair specific characteristic, X, while a negative value may suggest potential disassortative sorting on this characteristic. Table 8 provides mean and standard deviation descriptives for this measure–i.e. comparing the summed pair values from the realized patient sharing collaboration pairs with counterfactual pairs obtained by switching physician partners. These results are in line with the results suggested by Table 2 within the main text, and further with the main estimation results of Table 3.

VARIABLE	MEAN	SD
cHospital11	1.73047	0.89524
cHospital12	1.73097	0.897079
cPractice11	1.05123	0.86589
cPractice12	1.05101	0.866732
dSystem11	1.09788	0.726149
dSystem12	1.098	0.726501
dGender11	0.0404606	0.749426
dGender12	0.0414289	0.74808
sExperience11	-27.5548	410.574
sExperience12	-29.1538	406.09
dSpecialty11	0.129094	0.682034
dSpecialty12	0.12631	0.685475
jSpecialty11	0.119815	0.528887
jSpecialty12	0.117024	0.532461
cMANetworks11	1.42703	4.59915
cMANetworks12	1.37597	4.67929
Distance11	-15.7929	14.3031
Distance12	-15.7738	14.3338
cOtherNetworks11	3.31117	5.05223
cOtherNetworks12	3.31509	5.05337

Table 8: Assortative (and Disassortative) Link Formation Check: Each variable represents a tetrad level between the realized and counterfactual values. That is, for a measure $X11$, we have: $X_{ij} + X_{kl} - (X_{ik} + X_{jl})$; and for a measure $X12$ we have: $X_{ij} + X_{kl} - (X_{il} + X_{jk})$.

Appendix B: Additional Specifications

A potential concern with the link specific marginal utility function used within the paper is that it may be misspecified. A finding that reduces this concern is the observation that the estimated model (presented within Table 3) is able to explain 98% of all tetrad level inequalities. To further support the viability of the main specification, I provide results from an alternative specification in Table 9. These results are for the link specific marginal utility specification:

$$mu_{ij} = \gamma cHospital_{ij} + X_{ij}^{'}\beta + A_i + A_j \tag{10}$$

$$+\delta\left(cHospital_{ij} * jSpecialty_{ij}\right) + \rho\left(cPractice_{ij} * jSpecialty_{ij}\right) + \xi_{ij}.$$

This specification provides additional controls for interactions between the hospital and practice affiliation measures and physicians' specialty overlap. The results from this alternative specification are provided within column (1) of Table 9. We see here that the results are qualitatively similar, and further that the additional benefit in terms of percentage of tetrad inequalities satisfied is minimal. This result highlights robustness of the main result of the paper and also provides some support for the more parsimonious specifications of the link specific utility explored within the main paper, as the improvement in terms of additional inequalities explained is small.

Link Channel	Variable	(1) Estimate	(2) Relative Contribution	(3) % Absolute Contribution
Hospital Affiliations	cSameHospital	33.869	46.4	39.4%
		(13.113, 36.463)		
Other Affiliations	cSamePractice	33.656	26.8	22.7%
		(12.951 , 38.089)		
	dSameSystem	8.255	7.5	6.4%
		(4.382, 11.321)		
Homophily	dGender	1	1	0.8%
		(-,-)		
	sExperience	-0.005	-1.4	1.2%
		(-0.008,-0.004)		
Specialty	jSpecialty	9.910	4.0	3.4%
		(3.672 , 11.458)		
Patient Considerations	cSameMA_Networks	0.484	1.4	1.2%
		(0.166, 0.592)		
	Distance	-1.541	-12.5	10.6%
		(-1.715, -0.858)		
Spillovers	cSameOther_Networks	1.134	26.2	22.2%
		(0.591, 1.414)		
Interactions	cPractice_jSpecialty	14.553	3.0	2.6%
		(3.363, 18.027)		
	cHospital_jSpecialty	-7.727	-2.8	2.4%
		(-10.185, 0.752)		
	Numb. Links	12,086	-	-
	Numb. Inequalities	115,668	-	-
	% Ineq. Satisfied	98.489	-	-
	Subsample Draws	100	-	-

Table 9: Maximum Score Estimates: A random sample 115,668 of all inequalities was used. 95% confidence regions were constructed using 100 random draws of a 30% subsample of physicians. The same gender dummy (*dGender*) is normalized to +1 and it is used as the reference parameter.

A second specification of the model is further explored in Table 10. This specification replaces the affiliation count variables ($cSameHospital_{ij}, cSamePractice_{ij}$) with corresponding dummy variables ($dSameHospital_{ij}, dSamePractice_{ij}$), and the specialty overlap ($jSpecialty_{ij}$) with the dummy variable ($dSpecialty_{ij}$). Here, for example, $dSameHospital_{ij} = 1\left(|H_i \cap H_j| > 0\right)$, where $1\left(.\right)$ is an indicator function and H_i denotes the set of hospitals that i is affiliated with. $dSamePractice_{ij}$ and $dSpecialty_{ij}$ are similarly defined. Looking at the signs of the effects and the overall relative magnitudes, the results from working with the dummies yield qualitatively similar results to those reported within the main text (Table 3). In terms of model fit, this specification is not as flexible as the specification used in the main paper, which is reflected in the marginally lower percentage of inequalities

satisfied by the dummy variable specification reported here.

		(1)	(2)	(3)
**Link Channel**	**Variable**	**Estimate**	**Relative Contribution**	**% Absolute Contribution**
Hospital Affiliations	dSameHospital	47.494<br>(26.530, 58.370)	56.4	51.4%
Other Affiliations	dSamePractice	48.567<br>(28.500, 60.741)	33.3	30.6%
	dSameSystem	5.583<br>(4.589, 7.952)	4.3	4.1%
Homophily	dGender	1<br>(-,-)	1	0.8%
	sExperience	-0.003<br>(-0.005, -0.002)	-1.0	0.8%
Specialty	dSpecialty	3.522<br>(1.307, 4.284)	2.1	1.1%
Patient Considerations	cSameMA_Networks	0.290<br>(0.171, 0.416)	0.8	0.7%
	Distance	-1.050<br>(-1.279, -0.795)	-8.5	6.7%
Spillovers	cSameOther_Networks	0.793<br>(0.566, 0.980)	18.3	14.5%
	Numb. Links	12,086	-	-
	Numb. Inequalities	115,668	-	-
	% Ineq. Satisfied	98.437	-	-
	Subsample Draws	100	-	-

Table 10: Maximum Score Estimates: A random sample 115,668 of all inequalities was used. 95% confidence regions were constructed using 100 random draws of a 30% subsample of physicians. The same gender dummy (*dGender*) is normalized to +1 and it is used as the reference parameter.
